# Novel Insights into the Biochemistry of Citrate and Its Transporters: Relevance to Metabolic Rewiring, Tumor Microenvironment, and Bone Metastasis in Cancer

**DOI:** 10.3390/cells15191811

**Published:** 2026-10-04

**Authors:** Vadivel Ganapathy, Sathish Sivaprakasam, Yangzom D. Bhutia, Puttur D. Prasad, Muthusamy Thangaraju

**Affiliations:** 1 Department of Cell Biology and Biochemistry, Texas Tech University Health Sciences Center, Lubbock, TX 79430, USA; sathish.sivaprakasam@ttuhsc.edu (S.S.); yangzom.d.bhutia@ttuhsc.edu (Y.D.B.); 2 Department of Biochemistry and Molecular Biology, Augusta University, Augusta, GA 30912, USA; pprasad@augusta.edu (P.D.P.); mthangaraju@augusta.edu (M.T.)

**Keywords:** citrate, SLC13A5, SLC25A1, itaconate, SLC13A3, hypoxia-inducible factors, iron homeostasis, lipid synthesis, cancer, bone metastasis, osteolysis

## Abstract

Citrate intersects multiple biochemical pathways, yet its role in cancer has received limited attention. Similarly, the Na^+^-coupled citrate transporter (SLC13A5) has been investigated extensively in connection with epileptic encephalopathy, but its relevance to cancer remains understudied. This review focuses on novel insights into citrate biology and SLC13A5 that suggest a tumor-promoting role for SLC13A5/citrate. Cytosolic citrate is the building block for fatty acid and cholesterol synthesis necessary for membrane biogenesis and serves as a signaling molecule in regulating mitophagy and proteostasis in the endoplasmic reticulum. Cytosolic citrate pools derived from SLC13A5-mediated uptake across the plasma membrane and SLC25A1-mediated export from mitochondria have distinct functions. Citrate inhibits asparaginyl hydroxylase and thus potentiates transcription via hypoxia-inducible factors, consequently enhancing lactatogenic aerobic glycolysis and glutaminolysis in cancer cells. Citrate chelates iron and zinc; hence, cancer cells may be protected from ferroptosis and subjected to metabolic alterations by citrate. Human SLC13A5 is coupled to Na^+^ and H^+^, a feature that accelerates the delivery of extracellular citrate into cancer cells in the acidic tumor microenvironment (TME). SLC13A5 is also expressed in intracellular vesicles where it sequesters cytoplasmic citrate, thereby relieving citrate-mediated inhibition of glycolysis. Because the bone matrix is rich in citrate, osteolytic bone metastasis may increase citrate levels in the TME, providing a citrate-rich niche that facilitates cancer cell growth, metabolic reprogramming, and resistance to ferroptosis. Collectively, this review highlights novel biological aspects of citrate and SLC13A5 as they relate to tumor growth and bone metastasis and identifies SLC13A5 as a potential therapeutic target in cancer.

## 1. Introduction

Citrate is a ubiquitous metabolite in almost every cell type. It is a widely recognized textbook knowledge that citrate is the key intermediate in the citric acid cycle (also known as the tricarboxylic acid cycle, TCA cycle or Krebs cycle) [1]. It is also equally well known that citrate, generated within the mitochondrial matrix, is transferred to the cytoplasm to serve as the source of acetyl-CoA for use as the building block in the synthesis of fatty acids, isoprenoids, cholesterol, dolichol, and ubiquinone [2,3]. There are, however, several other, relatively lesser-known, functions of this metabolite that are now being recognized as biologically important not only in normal tissue but also in cancers and cancer-associated stroma [4,5]. This includes the role of citrate as a signaling and regulatory molecule, and multiple citrate transporters expressed in different locations/organelles of specific cell types that mediate citrate transfer from one site to another within the cell for distinct biological functions and for citrate import into and citrate release out of the cells for transcellular transfer and cell-to-cell communication [5,6,7,8,9,10]. New insights are emerging from recent investigations that imply a tumor-promoting role for citrate and its transporters. The focus of this review is to highlight these relatively less recognized and less appreciated new findings in the biology of citrate and its transporters, particularly their connection to cancer.

## 2. Biochemistry and Cellular Functions of Citrate

Citrate is a tricarboxylic acid; the dissociation constants (pKa) for its three carboxylic acid groups are 3.1, 4.3, and 5.6. At a normal physiological extracellular pH (i.e., 7.4), the fully deprotonated form (i.e., trivalent citrate^3−^) predominates (~90%), with the remainder being the monohydrogen citrate (i.e., divalent citrate^2−^); the dihydrogen citrate (i.e., monovalent citrate^−^) is negligible. The trivalent form is even more pronounced within the mitochondrial matrix, where pH is around 8.3 (citrate^3−^, ~99%). The trivalent and divalent ionic forms of citrate highlight its role as a metal chelator. Citrate can chelate trivalent (e.g., Fe^3+^) and divalent (e.g., Fe^2+^, Ca^2+^, Zn^2+^, and Mg^2+^) metal ions. The ability of citrate to bind Ca^2+^ is the basis for its function as an anticoagulant and as a structural component in the bone matrix. Calcium is needed for the normal function of the four vitamin-K-dependent clotting factors that contain γ-carboxyglutamate residues whose side chains possess two carboxylate groups to interact with the divalent Ca^2+^. Citrate competes with these clotting factors for chelation with Ca^2+^, thus interfering with the functions of these factors in the clotting cascade. The chelation of Ca^2+^ by citrate also helps to prevent kidney stones by interfering with the formation of calcium oxalate and calcium phosphate crystals. Bone contains high levels of citrate, which constitutes approximately 5% of its organic matter. In this tissue, citrate binds to Ca^2+^ on the surface of apatite crystals and stops bone minerals from thickening too much, thereby preventing brittle bones and providing resistance to fractures [11]. Recently, we reported on the development of a simple in vitro technique to screen for new iron chelators based on the ability of radiolabeled citrate to bind Fe^3+^ [12].

Within the mitochondria, citrate initiates the TCA cycle and generates NADH and FADH_2_, the two major electron donors for the electron transport chain. In this process, two of the three carboxylate groups in citrate are converted to CO_2_ and the remaining four carbon atoms end up as oxaloacetate. Subsequently, the electrons in NADH and FADH_2_ are transferred to molecular oxygen via the electron transport chain to set up a transmembrane H^+^ gradient across the inner mitochondrial membrane, which then serves as the driving force for ATP synthesis via F_0_F1-ATPAse (a misnomer for ATP synthase). When needed, as dictated by the metabolic status of the cell, citrate is transported out of the mitochondria into the cytoplasm where it is cleaved by ATP-citrate lyase to produce acetyl-CoA and oxaloacetate, with subsequent funneling of acetyl-CoA through anabolism to synthesize fatty acids, isoprenoids, cholesterol, ubiquinone, and dolichol. Oxaloacetate, on the other hand, can be converted to malate via malate dehydrogenase, which then acts as a substrate for the malic enzyme to produce NADPH, the reducing power necessary for fatty acid/isoprenoid/cholesterol synthesis. In addition to serving as the source for the carbon backbone for fatty acids, isoprenoids, and cholesterol, acetyl-CoA is also the substrate for protein acetylation in the cytoplasm, nucleus [13,14], and endoplasmic reticulum (ER) [15,16], with a significant impact on metabolic homeostasis and ER-associated protein quality control.

Citrate also plays a regulatory role in the control of glycolysis, gluconeogenesis, and fatty acid synthesis by direct interaction as an allosteric modulator with specific enzymes involved in these biological processes. Phosphofructokinase-1 is one of the rate-limiting enzymes in glycolysis, and citrate inhibits this enzyme via allosteric interaction. Fructose-1,6-bisphosphatase is a key enzyme in gluconeogenesis that reverses the reaction mediated by phosphofructokinase-1; citrate is an allosteric activator of fructose-1,6-bisphosphatase. Thus, citrate suppresses glycolysis and activates gluconeogenesis by serving as a modulator of key enzymes to influence the direction of the following chemical reaction: fructose-6-phosphate + ATP ↔ fructose-1,6-bisphosphate + AMP; this reaction is driven in the forward direction by phosphofructokinase-1 (glycolysis) and in the reverse direction by fructose-1,6-bisphosphatase (gluconeogenesis). There is an additional mechanism by which citrate regulates glycolysis and gluconeogenesis; this involves the control of cellular levels of fructose-2,6-bisphosphate, an important metabolite that activates phosphofructokinase-1 and inhibits fructose-1,6-bisphosphatase. This molecule is produced from fructose-6-phosphate by phosphofructokinase-2, a component of the bifunctional enzyme phosphofructokinase-2/fructose-2,6-bisphosphatase; citrate inhibits phosphofructokinase-2, thereby decreasing the generation of fructose-2,6-bisphosphate. This also contributes to the citrate-mediated inhibition of glycolysis and activation of gluconeogenesis. With regard to fatty acid synthesis, citrate not only serves as the source of acetyl-CoA in the cytoplasm (generated by ATP-citrate lyase) but also functions as an allosteric activator of acetyl-CoA carboxylase, the rate-limiting enzyme in fatty acid synthesis that converts acetyl-CoA to malonyl-CoA as the substrate for the fatty acid synthetase complex (FASN). Malonyl-CoA is also an inhibitor of carnitine-palmitoyl transferase-1, a key enzyme necessary for the transfer of fatty acids into a mitochondrial matrix for subsequent oxidation via β-oxidation. Therefore, citrate coordinately promotes fatty acid synthesis in the cytoplasm and suppresses fatty acid oxidation in the mitochondrial matrix: citrate-induced activation of acetyl-CoA carboxylase increases malonyl-CoA production, providing substrate for fatty acid synthesis while simultaneously inhibiting carnitine-palmitoyl transferase-1 to prevent the entry of fatty acids into the mitochondria for subsequent oxidation.

The biological functions of citrate described above have all been well recognized for several decades. However, there are several lesser-known functions of citrate which are as important, if not more, as those listed in the previous paragraphs that have not received the attention they deserve. For example, citrate plays an essential role as a signaling molecule to control anabolic/catabolic pathways and cell proliferation. Importantly, the signaling cascades influenced by citrate are key determinants of a wide spectrum of biological processes not only in normal cells but also in cancer cells. The unique tissue distribution of citrate with >80% of the body’s pool residing in the bone [17] has also not received much attention, particularly in relation to the proliferation of metastatic cancer cells within this tissue. Furthermore, recent studies have suggested the potential involvement of citrate in the control of immune function [18]. Itaconate is an immunometabolite generated in macrophages, neutrophils, osteoclasts and tumor cells, where it plays a broad spectrum of autocrine and paracrine functions in the control of inflammation. This important molecule is synthesized from *cis*-aconitate, a transient intermediate in the conversion of citrate to isocitrate, in the citric acid cycle [19,20]. Citrate is also involved in the generation of the oncometabolite 2-hydroxyglutarate in the mitochondria and cytoplasm when the mitochondrial isocitrate dehydrogenase-2 (IDH2) and the cytosolic isocitrate dehydrogenase-1 (IDH1) are mutated, as in certain cancers [21].

## 3. Citrate Transporters

### 3.1. SLC25A1 (CIC)

**Figure 1:**
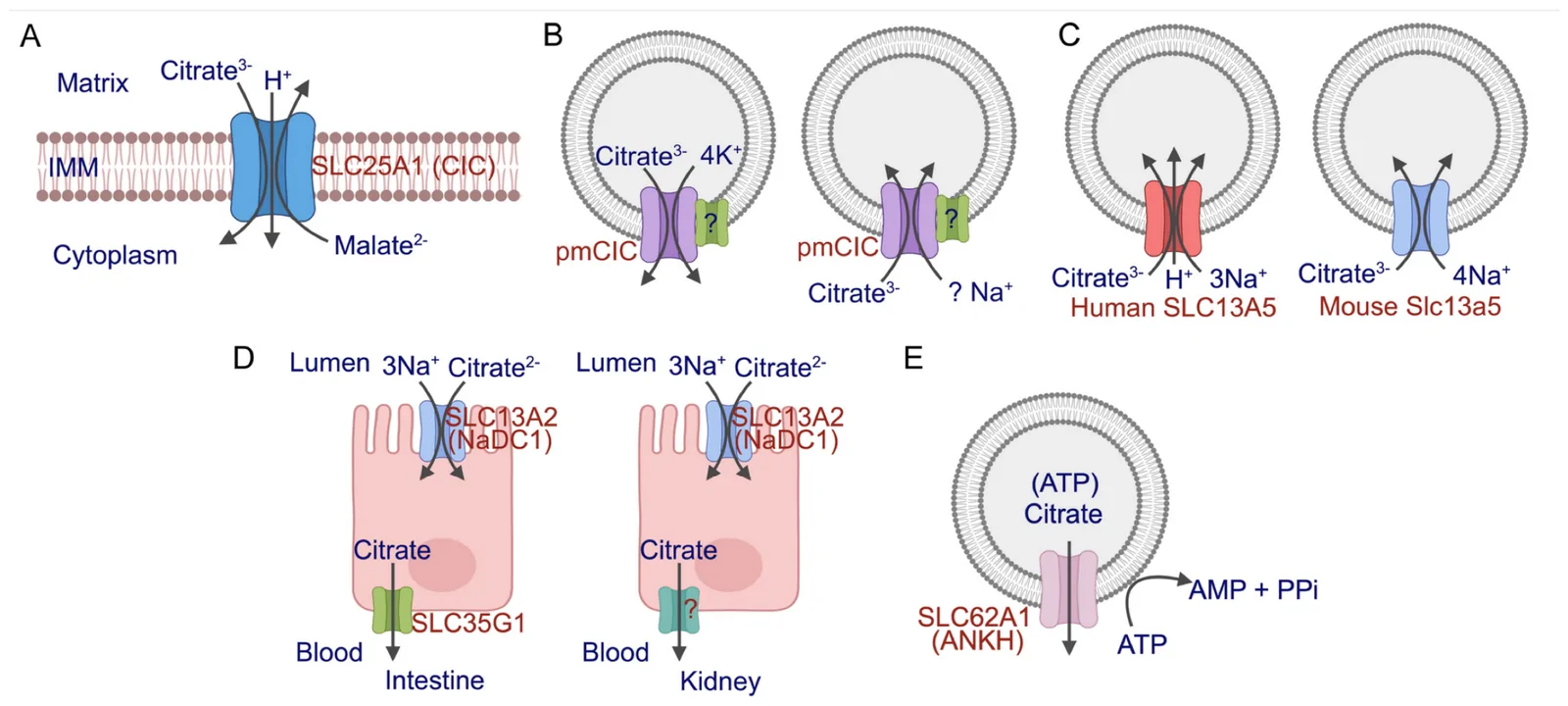
Citrate transporters in mammalian cells and their transport mechanisms. IMM, inner mitochondrial membrane; CIC, citrate/isocitrate carrier; pmCIC, plasma membrane citrate/isocitrate carrier; NaDC1, Na^+^-coupled dicarboxylate transporter 1; ANKH, human homolog of mouse ankylosis gene product. Question marks indicate that the relevant information has not yet been deduced.

The endogenous synthesis of citrate occurs exclusively within the mitochondrial matrix by citrate synthase, the first enzyme in the citric acid cycle, using oxaloacetate and acetyl-CoA as precursors. Citrate then goes through the cycle to regenerate oxaloacetate, one of the starting substrates, at the end of the cycle, and produces CO_2_, NADH, FADH_2_, and GTP during the cycle. NADH and FADH_2_ are used in oxidative phosphorylation involving the electron transport chain and F_0_F_1_-ATPase (actually, it is an ATP synthase) to generate ATP. When ATP levels rise within the cell, it suppresses the citric acid cycle by inhibiting key enzymes in the cycle as a feedback mechanism to maintain cellular energy status. Under these conditions, citrate is transported out of the mitochondrial matrix instead of getting broken down in the citric acid cycle. This transfer across the inner mitochondrial membrane requires the transporter SLC25A1. This transporter is commonly known as the mitochondrial citrate transport protein (CTP) or CIC (i.e., citrate/isocitrate carrier) because it accepts not only citrate but also isocitrate as its substrate [22]. Even though SLC25A1 resides in the mitochondria, it is encoded by the nuclear genome. It is a hetero exchanger because the transport process involves the export of citrate (or isocitrate) from the mitochondrial matrix, which is obligatorily coupled to the influx of malate (or succinate or phosphoenolpyruvate) into the matrix (Figure 1A). Only the divalent form of citrate (citrate^2−^) is accepted as the substrate and therefore the transporter is an electroneutral hetero exchanger for dicarboxylates (citrate^2−^ out and malate^2−^ in). There is no involvement of Na^+^ in the transport process. At a normal mitochondrial pH, which is approximately 8.3, citrate exists almost entirely in its trivalent form; therefore, mechanistically, the transport process mediated by SLC25A1 involves export of one H^+^ along with citrate^3−^ (i.e., citrate^3−^ + H^+^ = citrate^2−^). At the normal cytosolic pH (~7.2), the exported citrate^2−^ is deprotonated to form citrate^3−^ and release H^+^. As a result, the transport process mediated by SLC25A1 in the transfer of citrate from the matrix to the cytoplasm could cause significant acidification of the cytosolic pH.

Based on the transport process mediated by SLC25A1 being an electroneutral hetero exchanger for citrate and malate, the transporter could work bidirectionally for citrate transfer across the inner mitochondrial membrane depending on the transmembrane gradients for the two substrates. Under physiological conditions, these gradients drive the transporter to function predominantly in the export of citrate from the mitochondria to the cytoplasm. Nonetheless, the ability of the transporter to function in the reverse direction, facilitating the entry of cytoplasmic citrate into the mitochondria, has been demonstrated in cancer stem cells [23]. In this case, SLC25A1 imports cytoplasmic citrate into mitochondria for subsequent metabolism in the citric acid cycle, thus promoting mitochondrial respiratory activity. This activity of SLC25A1 in the reverse direction still involves hetero exchange, where the transfer of citrate^2−^ from the cytoplasm to the mitochondrial matrix is coupled to the transfer of malate^2−^ in the opposite direction.

SLC25A1 was first cloned from rat liver by Kaplan et al. [24]; the human homolog was cloned later by Goldmuntz et al. [25] and Iacobazzi et al. [26]. The mature human transporter consists of 298 amino acids with six transmembrane domains. The sequence of the mature protein is preceded by a 13-amino-acid sequence that helps to target the protein to mitochondria.

### 3.2. pmCIC

An alternatively spliced human SLC25A1 was cloned and functionally characterized by Mazurek et al. [27]. In contrast to SLC25A1, this splice variant localizes to the plasma membrane instead of the inner mitochondrial membrane, and therefore is referred to as pmCIC (i.e., plasma membrane citrate/isocitrate carrier). SLC25A1 and pmCIC differ only in the first exon, with the remaining exons 2–9 being identical for both. The mature pmCIC consists of 318 amino acids with an *N*-terminus sequence that is distinct from that of the mature SLC25A1. The *N*-terminus of pmCIC possesses multiple cationic amino acids, which have been hypothesized to be involved in the trafficking of this splice variant to the plasma membrane [27], but direct experimental evidence in support of this hypothesis is lacking. Functional analysis in heterologous expression systems revealed that pmCIC mediates the K^+^-dependent electrogenic release of citrate from the cells. This splice variant is expressed in normal prostate epithelial cells. These cells are known to release large amounts of citrate into prostatic fluid, and the functional features of the transport process responsible for this phenomenon are identical to those of the cloned pmCIC [27,28]. This indicates that the splice variant is responsible for citrate release in prostate epithelial cells. Interestingly, pmCIC does not function as a hetero exchanger and there is no involvement of counter transport of malate, as seen with SLC25A1 [27]. This is a surprising distinction given that the two proteins differ only in their *N*-terminal sequence (30 amino acids in SLC25A1; 37 amino acids in pmCIC) but are identical in the sequence of the remaining portion (281 amino acids). It has been speculated that pmCIC may work with an auxiliary protein in the plasma membrane to impart these significant variations (K^+^ dependence, electrogenicity, non-involvement of hetero exchange with malate) in the transport mechanism (Figure 1B), but the identity of no such interacting protein has been established experimentally. Another interesting twist is the ability of pmCIC to mediate Na^+^-dependent influx of citrate into cells, particularly in heterologous expression systems [27] and metastatic cancer cells (Figure 1B) [29]. It is unclear whether the findings that a voltage-gated Na^+^ channel regulates Na^+^-dependent citrate entry into metastatic cancer cells, which do not express any other Na^+^-coupled citrate transporters known to date [30], have any relevance to the Na^+^-dependent influx of citrate mediated by pmCIC. As such, the possible involvement of other protein components in the cell-type-specific modification of the transport modalities of pmCIC remains only a hypothesis at present; there is no experimental data on the identity of any of these postulated auxiliary proteins.

### 3.3. SLC13A5 (NaCT)

SLC13A5, also known as sodium-coupled citrate transporter (NaCT), is the only unequivocal Na^+^-coupled transporter with high specificity for citrate in mammalian cells. It was first cloned from rat brain [31], followed by human [32] and mouse [33] homologs. In all three cases, the transport function is obligatorily coupled to Na^+^, and the transport process is electrogenic. The rodent Slc13a5 exhibits significantly higher affinity for citrate (*K*_t_, ~30 μM) than human SLC13A5 (*K*_t_, 0.7–5.0 mM). As such, the rodent Slc13a5 is described as the high-affinity/low-capacity type, whereas the human SLC13A5 is described as the low-affinity/high-capacity type. The marked difference in citrate affinity between the rodent and human SLC13A5s is physiologically important because at normal plasma citrate concentrations (~150 μM), the rodent transporter is expected to be saturated, whereas the human transporter is not. This means that if the concentration of citrate increases above the physiological level, there will be no corresponding increase in transport activity in the case of the rodent transporter; in contrast, the transport activity of the human transporter will respond positively to increases in citrate levels.

The affinity of human SLC13A5 for citrate seems to vary markedly depending on the experimental conditions. When the affinity is determined for the newly expressed human SLC13A5 in heterologous expression systems, the *K*_t_ value is ~0.7 mM [32], whereas when the native transporter expressed in human liver cell line HepG2 is used as the experimental system, the value is almost ten times higher (~5 mM) [34]. This marked difference cannot be discounted simply based on technical variations. The exact reasons for these varied affinity values for citrate remain unexplored. A potential mechanism underlying this difference may involve post-translational modifications of the transporter. When the heterologous expression system is used for affinity determination, the transporter is a newly synthesized protein, and it may not have undergone post-translational modifications (e.g., acetylation and/or phosphorylation). In contrast, the constitutively expressed transporter in HepG2 cells might have been modified by such post-translational modifications, which could potentially impact substrate affinity. This explanation is purely speculative at present; its validity remains to be assessed by actual experimentation and biochemical analysis of the transporter protein under the two experimental conditions.

Another important difference between rodent and human SLC13A5s is their response to Li^+^: the rodent transporter is inhibited by Li^+^, whereas the human transporter is stimulated by Li^+^ [35,36]. These effects of Li^+^ are seen in both cases only when their transport activities are monitored in the presence of Na^+^; there is no measurable transport activity in both cases when Na^+^ is absent, indicating that Na^+^ cannot be substituted completely by Li^+^. The two homologs also differ in their responses to extracellular pH and transmembrane H^+^ gradient. The transport activity of the rodent Slc13a5 is not affected by extracellular pH in the range of 5.5 to 8.5, whereas the human SLC13A5 is markedly stimulated by acidic pH. Furthermore, the transport of citrate via the rodent Slc13a5 has no effect on intracellular pH; in contrast, the transport of citrate via the human SLC13A5 leads to intracellular acidification. These findings indicate that the transport process does not involve cotransport of H^+^ in the case of rodent Slc13a5, and that since the transport is not affected by external pH, the transporter accepts only the trivalent citrate anion (citrate^3−^) as the substrate. The electrogenic nature of the process suggests a Na^+^:citrate^3−^ stoichiometry of 4:1. In contrast, the human SLC13A5 cotransports the divalent citrate anion (citrate^2−^) with H^+^ coupled to Na^+^ symport. The electrogenic nature of the transport process therefore indicates a likely Na^+^:H^+^:citrate^2−^ stoichiometry of 3:1:1. This would provide a reasonable explanation for the stimulatory effect of acidic external pH and the acidification of intracellular pH. It is also likely that at least one, not all, of the Na^+^-binding sites can accept Li^+^ with a much higher affinity than Na^+^, thus explaining why Li^+^ increases the transport activity but only in the presence of Na^+^. When the human transporter is activated by H^+^ or Li^+^, the affinity of the transporter for citrate increases. These complex differential interactions of H^+^ and Li^+^ with rodent and human SLC13A5s are illustrated in Figure 1C.

### 3.4. SLC13A2 (NaDC1) and SLC35G1

SLC13A2, also known as Na^+^-coupled dicarboxylate transporter 1 (NaDC1), is a low-affinity transporter for various intermediates in the citric acid cycle; its transport function is obligatorily dependent on Na^+^ and its substrates are dicarboxylates such as succinate, fumarate, and malate. Citrate also serves as a substrate for this transporter, but only in its divalent form (citrate^2−^). NaDC1 was first cloned from rabbit kidney [37]; subsequently, mouse, rat and human homologs were cloned [38,39,40]. This transporter is expressed predominantly in the kidney and the intestinal tract, where citrate is known to be reabsorbed from the glomerular filtrate (kidney) or absorbed from exogenous sources such as diet or bacterial metabolism (small intestine). The localization of NaDC1 in the polarized epithelial cells in the kidney and small intestine is restricted to the lumen-facing apical membrane, meaning that the transporter is responsible for the first step in the (re)absorption of citrate by mediating the entry of citrate from the lumen into the cells (Figure 1D). The transport process is electrogenic, with a Na^+^:citrate^2−^ stoichiometry of 3:1. The preference of the divalent form of its substrates is evident from the findings that external pH has little effect on succinate transport, whereas citrate transport is markedly stimulated by external acidic pH. The affinity of the transporter for citrate is in the low millimolar range (*K*_t_, 0.6–1.2 mM). As citrate, an effective calcium chelator, prevents the formation of calcium oxalate and calcium phosphate crystals, the handling of citrate by NaDC1 in the kidney has clinical significance in relation to nephrolithiasis [41,42].

Intestinal absorption and renal reabsorption of citrate are transcellular processes involving citrate entry from the lumen into epithelial cells across the apical membrane, followed by its exit from the cells into the blood across the basolateral membrane. Recently, Mimura et al. [8] have identified SLC35G1 as the transporter responsible for the transfer of citrate across the intestinal basolateral membrane. Among the intermediates of the citric acid cycle, only citrate serves as the substrate for this transporter (*K*_t_, ~0.5 mM). It is interesting that even though SLC35G1 accepts only the divalent form of citrate (citrate^2−^) as its substrate, other dicarboxylates such as succinate, malate and fumarate are excluded as substrates by the transporter. The transport mechanism for SLC35G1 is distinct from that for SLC13A2 (NaDC1) (Figure 1D). SLC35G1 is a Na^+^-independent facilitative transporter. Sensitivity to Cl^−^ ions is a unique feature of this transporter that has significant physiological relevance for its function as an exporter of citrate from the cells into blood. In theory, a facilitative transporter should be able to function bidirectionally, with the direction of substrate movement dictated solely by the direction of the transmembrane gradient for the substrate. In the case of SLC35G1, transport of citrate into cells is almost completely inhibited by extracellular Cl^−^ at physiological concentrations. However, as intracellular levels of Cl^−^ are much lower (~5 mM) than the Cl^−^ levels in blood (~120 mM), SLC35G1 transfers citrate across the basolateral membrane preferentially in the cell-to-blood direction, with the re-entry of citrate into cells via the transporter blocked by Cl^−^. As such, a functional synergy between the apical SLC13A2 and the basolateral SLC35G1 enables movement of citrate from the intestinal lumen to the blood (Figure 1D). There is no evidence for the expression of SLC35G1 in renal tubular cells. As such, the transporter responsible for the exit of reabsorbed citrate from the cells into blood remains unknown.

### 3.5. SLC62A1 (ANKH)

*Ank* is the ankylosis gene in mice that codes for a membrane protein with multiple transmembrane domains, and *ANKH* is the corresponding human homolog. Loss-of-function mutations or gain-of-function mutations in this gene in humans cause distinct types of skeletal mineralization diseases [43]. For a long time, the protein coded by ANKH was thought to be involved in the export of pyrophosphate from the cells [44]. However, a recent study has shown that the protein transports ATP, not pyrophosphate, from the cells and that the released ATP is almost immediately hydrolyzed extracellularly to generate AMP and pyrophosphate [9]. Directly relevant to the current review is the discovery that the protein also mediates the release of citrate, malate and succinate (Figure 1E) [9]. Even though very little is known about the transport mechanism in terms of ionic dependence, substrate affinity, and the role of membrane potential, the protein’s role in citrate export from the cells is evident from the findings that the plasma concentration of citrate is reduced to 25% of the control value in mutant mice that lack Ank activity [9]. Similarly, bone citrate levels are decreased by 50% in mutant mice. Extracellular citrate plays an essential role in bone biology. Osteoblasts are known for their robust capacity for endogenous citrate production and for SLC13A5-mediated uptake of citrate from blood; as such, it is likely that ANKH is responsible for the release of citrate from these cells to support bone mineralization and stabilization of hydroxyapatite crystals, as proposed recently [45]. Based on the convincing evidence that ANKH is indeed a transporter, it is now identified by the systematic name SLC62A1.

## 4. Genetic Diseases Associated with Loss-of-Function Mutations in Citrate Transporters

Mutations (or single-nucleotide polymorphisms) that alter the activity of any of the four citrate transporters described above cause diseases with significantly different clinical sequelae resulting from disrupted citrate homeostasis in affected tissues. Defective function of the mitochondrial citrate exporter SLC25A1 is involved in the pathogenesis of either the more severe combined D-2/L-2-hydroxyglutaric aciduria [46] or the relatively milder congenital myasthenic syndrome type 23 [47]. The severe form results from almost a complete lack of transport function of SLC25A1, and the clinical features of the disease include neonatal encephalopathy, developmental delay, neuromuscular deficit, hypotonia, respiratory problems, and early death. The milder form, resulting from partially defective function of the transporter, is associated with early onset muscle weakness, problems in breathing, difficulty in swallowing, and drooping eyelids, all related to a deficiency in neuromuscular transmission. The common involvement of SLC25A1 in the etiology of both diseases is supported by the presence of significant overlap in clinical consequences between them. As the primary function of SLC25A1 is to export citrate out of the mitochondrial matrix, defective function of this transporter increases the levels of citrate within the matrix and at the same time decreases cytoplasmic citrate. One of the downstream metabolites of citrate within the mitochondria is α-ketoglutarate, which accumulates because of defective export of citrate from the mitochondria and is transferred to the cytoplasm via multiple dicarboxylate carriers in the inner mitochondrial membrane. The pathologically elevated levels of α-ketoglutarate are then funneled through lactate dehydrogenase and malate dehydrogenase to generate the L-isomer of 2-hydroxyglutarate, and through phosphoglycerate dehydrogenase to generate the D-isomer of 2-hydroxyglutarate [48], which are excreted in urine, resulting in D-2/L-2-hydroxyglutaric aciduria. The decrease in cytosolic citrate likely leads to defective synthesis of fatty acids, sterols, dolichol, and ubiquinone in a tissue-specific manner, including the brain and muscle. Cytosolic citrate is also the source of acetyl-CoA for the synthesis of the neurotransmitter acetylcholine; as such, a decrease in cytoplasmic citrate may lead to defective formation of this signaling molecule, leading to a neuromuscular deficit. Furthermore, both isomers of 2-hydroxyglutarate function as important signaling molecules by serving as competitive inhibitors of several α-ketoglutarate-dependent dioxygenases; this has a profound impact on DNA and protein methylation, thus markedly influencing gene transcription. Therefore, the increased levels of these molecules could contribute to the etiology of the clinical phenotype of both disease types. This view is supported by the findings that citrate supplementation has beneficial effects in patients with D-2/L-2-hydroxyglutaric aciduria and that patients with myasthenic syndrome show a better clinical outcome when treated with mild inhibitors of acetylcholine esterase, which improve acetylcholine-mediated neurotransmission [49,50].

Citrate in the glomerular filtrate in nephrons inhibits the formation of calcium oxalate and calcium phosphate stones in the kidney. The Na^+^-coupled dicarboxylate transporter NaDC1 (SLC13A2), expressed primarily in the lumen-facing apical membrane of absorptive cells in the kidney, controls the amount of citrate in urine by facilitating the reabsorption of filtered citrate. Because of the activity of citrate as a calcium chelator, hypocitraturia increases the risk of renal stones, whereas increased citrate in urine protects against renal stones. Single-nucleotide polymorphisms in the gene coding for SLC13A2 have been shown to be connected to nephrolithiasis, both as a facilitator or a protector of renal stone formation, depending on whether a given polymorphism decreases or increases the activity and/or expression of the transporter [42]. This is also the molecular basis for the promotion of kidney stones in metabolic acidosis because systemic acid pH increases the expression and function of SLC13A2 in the kidney [51]. By the same logic, there may also be a synergistic interaction between changes in the activities of the chloride/oxalate exchanger SLC26A6, which controls oxalate levels in the nephron lumen, and the citrate transporter SLC13A2, which controls citrate levels in the nephron lumen [52].

Loss-of-function mutations in SLC13A5 cause a devastating disease known as early infantile epileptic encephalopathy-25 (EIEE-25) [53,54,55,56]. It is primarily a neurological disorder with severe clinical symptoms including frequent seizures, skeletal–muscular developmental problems, behavioral and developmental delays, and speech difficulties. In contrast to humans with loss-of-function mutations in SLC13A5 with this severe disease, mice lacking this transporter show no obvious neurological symptoms but rather a beneficial phenotype with protection against diet-induced obesity and metabolic syndrome [57,58]. We have hypothesized that the marked differences in transport function between human SLC13A5 and mouse Slc13a5 might be responsible for this profound difference in clinical phenotype resulting from the absence of the transporter [7,45,59,60]. It seems that the original name for the *Drosophila* homolog of SLC13A5, namely the longevity gene I’m Not Dead Yet (INDY), may be apt only for the transporter in non-human species. Although the exact mechanism underlying the neurological dysfunction and development in humans due to SLC13A5 deficiency remains largely unknown, several theories, including the role of citrate as a high-energy metabolite, a zinc chelator, a modulator of protein acetylation, and a precursor for the neurotransmitters acetylcholine, glutamate and γ-aminobutyrate, have been proposed, but none has been established unequivocally as the major etiologic factor in EIEE-25.

Loss-of-function mutations in the citrate/ATP exporter SLC62A1 (ANKH) that interfere with its transport function cause the mineralization disorder craniometaphyseal dysplasia [43]. As mentioned earlier in this review, SLC62A1 was thought to be an exporter for pyrophosphate, which could explain the pathogenesis of the disorder due to increased levels of pyrophosphate in extracellular fluids in joints. However, recent studies have shown that ANKH is an exporter for citrate and ATP, the latter of which is efficiently hydrolyzed to AMP and pyrophosphate following ANKH-mediated export, thus explaining the decrease in extracellular levels of pyrophosphate and citrate in humans with loss-of-function mutations in this transporter. Citrate and pyrophosphate act as inhibitors of calcium hydroxyapatite crystal formation in joints. Therefore, the decreased export of citrate and ATP due to ANKH deficiency promotes crystal formation and results in inflammation and bony fusion of the joints. In contrast, mutations in ANKH that activate its transport function increase ATP export and thus elevate extracellular levels of pyrophosphate; this has been shown to be the etiologic factor in the pathogenesis of certain forms of skeletal disorders associated with increased deposition of calcium pyrophosphate crystals in bones [43]. It is not known whether the accompanying increased export of citrate plays any role in the genesis of pathological features of these diseases.

## 5. Differential Functions of Cytoplasmic Citrate Depending on Its Origin via SLC25A1 or SLC13A5

There are only two sources of cytoplasmic citrate in mammalian cells: export from the mitochondrial matrix via SLC25A1 and import from the extracellular milieu via SLC13A5. The involvement of SLC13A2 (NaDC1) in the import of citrate into cells is discounted in this context because this process is principally restricted to the intestine and kidney to facilitate transcellular transfer of citrate from one side of the cell to the other. It is generally assumed that SLC25A1-mediated transfer from the mitochondria is the major determinant of citrate levels in the cytoplasm and that SLC13A5-mediated import of citrate may become a significant contributor only under conditions of hypoxia and glutamine deprivation [61]. These observations were made in human liver cancer cells and in rat neurons. It remains to be seen whether this is true in normal cells in humans. Once within the cytoplasm, irrespective of its origin, citrate is cleaved by ATP-citrate lyase to generate acetyl-CoA and oxaloacetate. Acetyl-CoA then goes through anabolic pathways to synthesize fatty acids and sterols and is also used as the donor of the acetyl group in protein acetylation.

**Figure 2:**
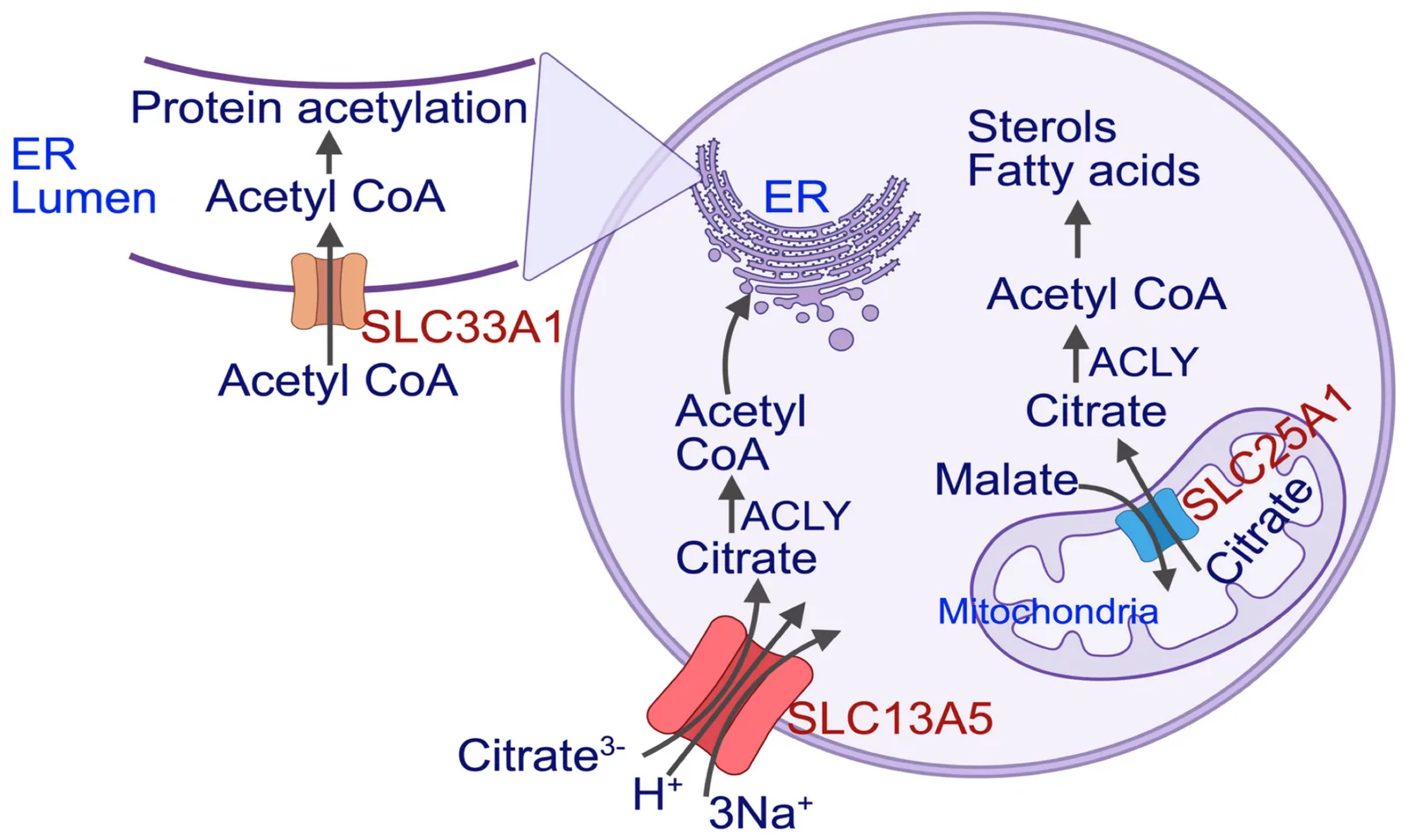
Differential cellular functions of citrate derived from the transport activity of the mitochondrial citrate transporter SLC25A1 and the plasma membrane citrate transporter SLC13A5. ER, endoplasmic reticulum; ACLY, ATP-citrate lyase.

Recent studies have uncovered interesting and biologically important new aspects of cytosolic citrate [62]. These studies have shown that the cytosolic citrate resulting from SLC25A1-mediated export from the mitochondria and SLC13A5-mediated import from extracellular sources represents distinct pools with access to different biochemical pathways (Figure 2). Cytoplasmic citrate is the primary source of acetyl-CoA, produced by the activity of ATP-citrate lyase, which is involved in fatty acid synthesis, sterol synthesis, and acetylation of proteins in the cytoplasm, nucleus, and endoplasmic reticulum. Interestingly, which of these pathways the cytoplasmic citrate is funneled through depends on whether it originates from the mitochondria or from the extracellular milieu.

The SLC25A1-generated citrate is utilized predominantly for fatty acid and sterol synthesis, whereas the SLC13A5-generated citrate is utilized for protein acetylation in the endoplasmic reticulum (ER). This distinction, however, is not absolute because there is some overlap in the use of citrate in different pathways, irrespective of its origin. Reversible protein acetylation is one of the key mechanisms by which gene expression (histone acetylation), protein stability and catalytic activity of specific metabolic enzymes are controlled. For example, acetylation of ATP-citrate lyase protects against ubiquitination and subsequent proteasomal degradation, thus enhancing its function in a feed-forward manner (i.e., acetyl-CoA is used to acetylate ATP-citrate lyase to increase the production of acetyl-CoA further). Acetyl-CoA is the starting material for fatty acid synthesis and sterol synthesis, where acetyl-CoA carboxylase participates in the former pathway and 3-hydroxy-3-methylglutaryl-CoA synthase-1 participates in the latter. These two enzymes are also regulated by acetylation. The catalytic activities of both enzymes are suppressed by acetylation, indicating negative feedback regulation in these cases. Cytoplasmic acetyl-CoA has free access to the nucleus to participate in histone acetylation as a mechanism for the control of gene expression. In the ER lumen, protein acetylation plays an obligatory role in quality control and proteostasis, and in the control of the secretory pathway [63]. Cytoplasmic acetyl-CoA does not have free access to the ER lumen and therefore a specific transporter (SLC33A1) is involved in the entry of cytoplasmic acetyl-CoA into this cellular compartment to participate in protein acetylation. In this context, it is interesting to note that the cytoplasmic citrate pool is not homogeneous. The cytoplasmic citrate arising from extracellular sources and imported into the cell via SLC13A5 is used preferentially to generate acetyl-CoA for protein acetylation in the ER lumen. In contrast, the cytoplasmic citrate arising from the mitochondrial source and imported into the cytoplasm via SLC25A1 is used preferentially to generate acetyl-CoA for the synthesis of fatty acids and sterols. However, there seems to be no significant difference in the use of cytoplasmic citrate as the source of acetyl-CoA for use in protein acetylation within the nucleus or cytoplasm. This new aspect of biologically distinct pools of cytoplasmic citrate is not just a biochemical curiosity; it has a profound impact on the whole organism. Overexpression of the plasma membrane SLC13A5 to increase the cytoplasmic pool of citrate originating from extracellular sources in mice leads to increased senescence and a progeria-like phenotype and reduces the lifespan of the animals significantly [62]. None of these effects is seen in mice when the mitochondrial SLC25A1 is overexpressed to increase the cytoplasmic pool of citrate originating from the mitochondrial matrix [62]. The mechanism that distinguishes the cytoplasmic citrate pool for differential use in protein acetylation in the ER lumen and in the anabolic pathways leading to fatty acid and sterol synthesis in the cytoplasm remains unknown at present.

The molecular mechanisms underlying the existence of biologically distinguishable pools of cytoplasmic citrate depending on its origin have not been elucidated. A potential mechanism might involve the formation of distinct metabolons consisting of specific molecular components with either SLC25A1 or SLC13A5. This strategy is known to work in several metabolic pathways to facilitate sequential substrate channeling from one enzyme to the next enzyme in a given metabolic pathway to reduce the risk of the metabolic intermediates drifting away from the enzymes. This increases the efficiency of the pathway. A similar mechanism might operate in the context of cytoplasmic citrate by incorporating the specific transporters along with relevant enzymes in the formation of metabolons (e.g., SLC13A5-ACLY-SLC33A1 metabolon versus SLC25A1-ACLY-FASN metabolon). This explanation for the different metabolically distinguishable pools of cytoplasmic citrate is only speculative at present, and its validation must wait for appropriate experimental studies.

A new function of cytoplasmic acetyl-CoA has been discovered recently, where it plays a key role in the control of mitophagy [64]. This involves the binding of acetyl-CoA to NOD-like receptor family member X1 (NLRX1), a protein associated with the outer mitochondrial membrane. When not bound to acetyl-CoA, NLRX1 interacts with light chain 3 (LC3) to facilitate the fusion of mitochondria with lysosomes and initiate mitophagy. The binding of acetyl-CoA to NLRX1 induces conformational changes in the protein such that its interaction with LC3 is prevented. As such, cytoplasmic acetyl-CoA protects mitochondria from mitophagy-mediated destruction. This interesting feature has been demonstrated only with the cytosolic acetyl-CoA originating from the mitochondrial source via SLC25A1-mediated export and with acetyl-CoA produced from acetate via the activity of acetyl-CoA synthetase short-chain family member 2. It is not known whether the cytoplasmic acetyl-CoA resulting from SLC13A5-mediated import of extracellular citrate has a similar ability to control mitophagy. Since the process involves the control of mitochondrial integrity, it is tempting to speculate that the observed control of mitophagy is selective for acetyl-CoA originating from mitochondria-derived citrate rather than for acetyl-CoA produced from SLC13A5-derived extracellular citrate, but this remains to be investigated.

Another biologically relevant and important new aspect about distinct pools of citrate in the cytoplasm is the recent discovery of the presence of SLC13A5 in cytoplasmic vesicles, where the transporter sequesters citrate within vesicles and thus reduces the levels of free citrate in the cytoplasm [65]. This has been shown thus far only in neurons; therefore, whether this mode of regulation of citrate pools in the cytoplasm occurs in other cell types remains to be seen. Nonetheless, the sequestration of cytoplasmic citrate in vesicles, as demonstrated in neurons, will have a profound impact on glycolysis, as citrate is a potent inhibitor of this metabolic pathway. The SLC13A5-mediated storage of cytoplasmic citrate within the vesicles will prevent citrate-mediated suppression of glycolysis, thus having a profound impact on cellular metabolism due to the resultant increase in glycolytic activity.

## 6. Cancer-Associated Changes in Cellular Levels of Citrate

### 6.1. Changes in the Expression of SLC25A1 and SLC13A5 in Cancer

Given the undisputed functions of cytoplasmic citrate in anabolic pathways (fatty acid and sterol synthesis), antioxidant machinery (NADPH production), control of glycolysis and regulation of gene expression and catalytic activities of important metabolic enzymes (protein acetylation), it is logical to predict a pro-cancer role for this metabolite. With the same logic, one might expect significant changes in the expression and function of the two transporters, SLC25A1 and SLC13A5, in cancer, as these transporters control the cytoplasmic levels of citrate. SLC25A1 and SLC13A5 could also control the levels of citrate within the mitochondrial matrix to participate in the citric acid cycle and thus stimulate oxidative phosphorylation, which will determine the energy status of the cell. The two transporters could also work sequentially to transfer extracellular citrate into the mitochondrial matrix, as SLC25A1 is freely reversible if the transmembrane concentration gradients for citrate and malate are favorable. Under normal conditions, the mitochondrial transporter SLC25A1 exhibits a widespread tissue distribution pattern; in contrast, the expression of the plasma membrane transporter SLC13A5 is limited only to a few tissues (liver, brain, testis). Accordingly, there are more reports in the published literature on SLC25A1 than on SLC13A5 in relation to cancer.

As per the publicly available cancer database (The Cancer Genome Atlas or TCGA), SLC25A1 is significantly upregulated in the following cancers: bladder cancer, esophageal cancer, glioblastoma, and cancers of the head and neck, kidney, liver, lung, and prostate [66,67]. The experimental evidence for this transporter in the promotion of cancer has been reviewed by Mosaoa et al. [5]; the reviewed studies have been done by evaluating the consequences of genetic or pharmacological inhibition of SLC25A1 in cancer cells, both in vitro and in vivo. The upregulation of SLC25A1 expression has been documented experimentally in triple-negative breast cancer [68,69,70], lung cancer [23,67,68], colorectal cancer [67,71], liver cancer [71,72,73], and prostate cancer [74]. In some cases, overexpression is associated with metastatic disease and poor patient prognosis, and cancer cell proliferation in vitro and tumor growth in xenografts in vivo are negatively affected by pharmacologic inhibitors of the transporter. Invariably, in most cases, the overexpression of SLC25A1 in cancer cells is associated with increased oxidative phosphorylation, lipid synthesis and antioxidant machinery. It may seem intuitively contradictory that increased export of citrate out of the mitochondrial matrix due to SLC25A1 overexpression promotes mitochondrial function in cancer cells. However, it should be borne in mind that citrate export is coupled to malate import, which could sustain the citric acid cycle and oxidative phosphorylation. Furthermore, cancer cells exhibit increased glutamine import and glutamine metabolism, where glutamine-derived α-ketoglutarate feeds into the citric acid cycle both in the forward direction to generate NADH and FADH_2_ for oxidative phosphorylation and in the reverse direction to generate citrate via reductive carboxylation (see below). Because of the excess production of citrate from glutamine metabolism within the mitochondria, the increased export of citrate via SLC25A1 will not interfere with mitochondrial function in cancer cells.

Several studies have demonstrated upregulation of the plasma membrane variant of SLC25A1, called pmCIC, in gastrointestinal, lung, prostate, pancreatic, urothelial, and skin cancers [10,29,75,76,77]. As detailed earlier in this review, the splice variant pmCIC promotes the entry of extracellular citrate into cancer cells across the plasma membrane. Interestingly, the same transporter may also be involved in the release of citrate into the tumor microenvironment from cancer-associated fibroblasts for subsequent uptake into tumor cells via the same transporter [75].

Unlike SLC25A1, the expression of SLC13A5 is relatively low in most tissues. The publicly available TCGA database indicates significant expression of SLC13A5 only in liver cancer, bladder cancer, pancreatic cancer, cervical cancer, lung cancer, and glioblastoma [78]. Among these, significant upregulation seems to occur only in pancreatic, bladder, cervical, and lung cancers. Thus far, detailed documentation of the involvement of SLC13A5 in cancer progression has been demonstrated only in liver cancer [79]. Genetic silencing of the transporter in human hepatocarcinoma cells leads to cell-cycle arrest and decreased cell proliferation in vitro and suppressed tumor growth in mouse xenografts in vivo. Interestingly, the TCGA database shows downregulation of this transporter expression in primary tumors in the liver [80].

### 6.2. Reductive Carboxylation in Cancer—Synthesis of Citrate from Glutamine

In normal cells, citrate is generated almost exclusively by citrate synthase in the mitochondrial matrix by the condensation of acetyl-CoA and oxaloacetate at the start of the TCA cycle. The citrate present in the cytoplasm results only from the export of the mitochondrial citrate via SLC25A1 and import of extracellular citrate via SLC13A5. However, there is an additional pathway for the generation of citrate, both in the mitochondrial matrix and in the cytoplasm, using carbons present in glutamine. It is widely recognized that cancer cells are “addicted” to glutamine [81,82] and exhibit a robust capacity to import extracellular glutamine via various glutamine transporters [81,82,83,84]. The metabolism of glutamine in cancer cells, called glutaminolysis, contributes not only to ATP production but also lactate production, in addition to the better-known functions of glutamine such as protein synthesis and nucleotide synthesis. In the context of the current review, the synthesis of citrate from glutamine is relevant. Generally, citrate is metabolized to isocitrate and α-ketoglutarate in the TCA cycle with the first two enzymes of the cycle, namely aconitase and isocitrate dehydrogenase (IDH). There are three isoforms of IDH: IDH1 is in the cytoplasm and IDH2 and IDH3 are in the mitochondrial matrix. The isoform that participates in the citric acid cycle in the forward direction is IDH3, which converts isocitrate to α-ketoglutarate; in this reaction, two electrons are added to NAD^+^ to produce NADH and one carboxylate group in isocitrate is removed as CO_2_. Because of the removal of electrons and CO_2_ from isocitrate, the reaction is called oxidative decarboxylation. In cancer cells, robust uptake and metabolism of glutamine generates high levels of α-ketoglutarate, which enters the TCA cycle to produce reducing equivalents NADH and FADH_2_ for ATP generation via oxidative phosphorylation and for lactate generation via the malate–malic enzyme–lactate dehydrogenase pathway. In contrast to IDH3, IDH1 and IDH2 could use not only NAD^+^/NADH but also NADP^+^/NADPH. When large amounts of α-ketoglutarate are made via glutaminolysis in cancer cells, IDH2 in the mitochondria functions in the reversal of the initial two steps of the TCA cycle along with aconitase. Using NADPH and CO_2_, IDH3 converts α-ketoglutarate to isocitrate, which is then converted to citrate by aconitase. As this pathway adds two electrons from NADPH to α-ketoglutarate and CO_2_ is used to create the third carboxylate group in isocitrate, the process is called reductive carboxylation. The same pathway also functions in the cytoplasm with IDH1. Thus, cancer cells generate mitochondrial and cytoplasmic citrate using glutamine as the precursor. The reductive carboxylation occurs prominently in cancer cells because of their marked capacity to take up and metabolize extracellular glutamine. This glutamine-derived citrate promotes cancer cell proliferation and tumor growth via its multiple biological functions.

There could be some biological connection between the overexpression of SLC25A1 and increased availability of glutamine in cancer cells that promotes reductive carboxylation. Experimental evidence indicates that the α-ketoglutarate/citrate ratio should be higher than normal to drive reductive carboxylation in the mitochondria [85]. This could be the case in cancer cells because increased expression of SLC25A1 would increase citrate export out of the mitochondria, and increased glutamine availability would increase the production of α-ketoglutarate, thus increasing the α-ketoglutarate/citrate ratio within the mitochondria. Furthermore, as reductive carboxylation requires NADPH, studies have shown that α-ketoglutarate going through the citric acid cycle in the forward direction generates NADH, a part of which is converted to NADPH by transhydrogenases [86]. What is interesting is the apparent synergism between the oxidation of a portion of the glutamine-derived α-ketoglutarate in the forward reactions of the citric acid cycle and the reduction of the rest of the glutamine-derived α-ketoglutarate in the reverse reactions of the citric acid cycle in fueling citrate synthesis from glutamine via reductive carboxylation in cancer cells.

## 7. Citrate as a Tumor Promoter—Role as a Metabolic and Signaling Molecule

### 7.1. Change in Cellular Energy Status as an Anabolic On-Switch in Response to Citrate

Citrate is an energy-rich metabolite; when it goes through the entire citric acid cycle, it generates 3 NADH and 1 FADH_2_, which serve as electron donors for the electron transport chain and establish the proton motive force (i.e., electrochemical H^+^ gradient) across the inner mitochondrial membrane. This osmotic energy is harnessed by the mitochondrial ATP synthase (F_0_F_1_-ATPase) to synthesize ATP from ADP. ADP is also used to produce ATP independent of oxidative phosphorylation via the enzyme adenylate kinase, which catalyzes the reaction: 2 ADP → AMP and ATP. The energy status of mammalian cells is described in terms of adenylate energy charge which is represented by the combined concentration of ATP and one-half of the concentration of ADP (in terms of cellular energy, 2 ADP is equivalent to 1 ATP, as per the adenylate kinase reaction). AMP is completely depleted of usable energy for the cells. As such, when cancer cells metabolize citrate in the citric acid cycle, ATP levels increase and AMP levels decrease. In a way, the concentration of AMP in mammalian cells can be considered as a biological indicator for the energy status of the cell in a reciprocal manner. The relative activities of the anabolic pathways and the catabolic pathways determine the energy status of the cell because the former utilize ATP and the latter generate ATP. Therefore, when cells are loaded with energy (i.e., high in ATP), anabolic pathways are promoted and catabolic pathways are suppressed. The reverse would occur when cells are low in energy (i.e., high in AMP). This intricate control is mediated at least in part via AMP-dependent protein kinase (AMPK) [87]. There is a reciprocal relationship between AMPK and mTOR (mechanistic target of rapamycin) in which AMPK phosphorylates mTOR, causing its inactivation. Thus, citrate could promote cancer by increasing the energy status of the cell, suppressing AMPK activity and consequently activating mTOR signaling, a well-established anabolic on-switch. This has been documented in liver cancer, where SLC13A5-mediated influx of extracellular citrate drives this pro-cancer signaling [79,88].

### 7.2. Control of the Transcriptional Activities of HIF-1α and HIF-2α by Citrate

The hypoxia-inducible factor HIF-1α is one of the key transcription factors involved in metabolic reprogramming in cancer cells. The expression and activity of this factor are controlled not only by hypoxia but also by mTOR, particularly the mTORC1 complex. As discussed in the previous section, citrate activates mTOR via suppressed AMPK activity. The resultant increase in mTORC1 signaling would increase the transcriptional activity of HIF-1α in cancer cells. The mTORC1-dependent increase in HIF-1α signaling involves three different mechanisms [89,90]: (i) an increase in the transcription of the HIF-1α gene; (ii) an increase in the translation of HIF-1α mRNA; and (iii) an increase in the transcriptional activity of HIF-1α.

In addition, there is another important mechanism by which citrate activates HIF-1α signaling. This involves the blockade of proteasomal degradation of HIF-1α protein via the inhibition of the HIF-1α hydroxylase FIH (factor inhibiting HIF-1; also known as asparaginyl hydroxylase) [91,92]. The ubiquitination and subsequent proteasomal degradation of HIF-1α are regulated by post-translational modification of the protein by hydroxylation of specific proline and asparagine residues, which is carried out by prolyl hydroxylases and asparaginyl hydroxylase, respectively. While the prolyl hydroxylases are subject to inhibition by metabolites such as fumarate, succinate, oxaloacetate, and citrate, the asparaginyl hydroxylase is inhibited primarily by citrate [91,92]. Oxaloacetate inhibits asparaginyl hydroxylase but with a potency much less than that of citrate; fumarate and succinate have no effect at all. This level of specificity to citric acid cycle intermediates as inhibitors is of interest given that prolyl hydroxylases, as well as the asparaginyl hydroxylase, are α-ketoglutarate-dependent dioxygenases. The resultant decrease in the hydroxylation of specific proline and asparagine residues interferes with the ubiquitination of HIF-1α protein, thus suppressing its degradation by proteasomes. As such, citrate potentiates HIF-1α signaling in cancer cells via specific inhibition of asparaginyl hydroxylase.

HIF-2α also plays a tumor-promoting role in cancer [93,94,95]. Recently, the FDA has approved an HIF-2α-selective inhibitor, known as Belzutifan, as an anti-cancer drug [96]. The signaling of HIF-2α is also regulated by asparaginyl hydroxylase, but by a mechanism distinct from its regulation of HIF-1α. Hydroxylation of specific asparagine residues in HIF-2α does not have any impact on the proteasomal degradation of the protein but suppresses its ability to function as a transcription factor [97]. Citrate, by inhibiting this hydroxylation, enhances the transcriptional activity of HIF-2α, thus providing another mechanism to promote cancer.

### 7.3. Aerobic Glycolysis and Glutaminolysis—Impact of Citrate-Induced Stabilization of HIF-1α

HIF-1α, along with c-MYC, is central to metabolic reprogramming in cancer cells. The salient features of cancer cell-specific metabolic changes are aerobic glycolysis (also known as the Warburg effect) and glutaminolysis. As citrate activates HIF-1α signaling, this metabolite plays a key role in setting up cancer cell metabolism towards aerobic glycolysis and glutaminolysis. We recently reviewed the most essential tenets of this metabolic reprogramming and discussed how these changes work synergistically with relevant nutrient transporters [82,84]. The term “aerobic glycolysis” is in some ways confusing because all this term refers to is glycolysis that occurs in the presence of oxygen; it does not differentiate between aerobic glycolysis that occurs in normal cells where glucose-derived pyruvate is oxidized to CO_2_ and aerobic glycolysis that occurs in cancer cells where glucose-derived pyruvate is converted to lactate. To avoid this confusion, we propose a new term to describe the aerobic glycolysis that is specific to cancer cells: lactatogenic aerobic glycolysis. This new term describes the two essential features of cancer-cell-specific glycolysis, namely occurrence in the presence of oxygen and conversion of glucose to lactic acid.

Enhanced glutamine metabolism in cancer cells is obligatorily coupled with increased import of extracellular glutamine via selective amino acid transporters [82,84]. Glutamine is converted to glutamate via glutaminases and then glutamate is converted to α-ketoglutarate via glutamate dehydrogenase. The resultant α-ketoglutarate is metabolized both within the mitochondria and in the cytoplasm because this metabolite crosses the inner mitochondrial membrane easily via several dicarboxylate transporters known to exist in this membrane. Within the mitochondria, α-ketoglutarate is metabolized in the citric acid cycle, both in the forward direction to generate NADH and FADH_2_ for oxidative phosphorylation to produce ATP and in the reverse direction to produce citrate via reductive carboxylation (see above). The reductive carboxylation to generate citrate from α-ketoglutarate also occurs in the cytoplasm with the cytoplasmic enzymes IDH1 and aconitase-1 (the corresponding mitochondrial enzyme is aconitase-2). Malate produced as an intermediate in the metabolism of α-ketoglutarate in the citric acid cycle in the forward direction crosses the inner mitochondrial membrane to enter the cytoplasm, where it is oxidatively decarboxylated by malic enzyme to generate pyruvate and NADPH, and the resultant pyruvate is then converted to lactate via LDHA. These multiple pathways of glutamine metabolism in cancer cells are collectively referred to as “glutaminolysis”. Contrary to the general assumption that tumor-associated lactic acid production originates exclusively from the lactatogenic aerobic glycolysis, glutaminolysis-derived lactic acid may actually constitute as much as 40% of the total lactic acid production in cancer cells [98,99,100]. Although this phenomenon has been documented only in a limited number of cancer cells (glioblastoma cells and K-RAS-dependent cancer cells), we hypothesize that the same phenomenon may occur in other cancer cells as well, provided they are “addicted” to glutamine, where glutamine is consumed more than what could be accounted for in its utilization in the synthesis of proteins and nucleotides.

### 7.4. Acute Versus Long-Term Effects of Citrate in Cancer Cells

Citrate is an allosteric inhibitor of PFK-1, the rate-limiting enzyme in glycolysis; this represents an acute effect. As PFK-1 is a cytoplasmic enzyme, the levels of citrate in this subcellular compartment dictate the extent of its action as an inhibitor of glycolysis. The expression and activities of the citrate transporters SLC25A1, pmCIC, and SLC13A5 in cancer cells control the cytoplasmic levels of this metabolite. The recent discovery of differential localization of the traditional plasma membrane transporter SLC13A5 in intracellular vesicles in excitable neurons [65] adds to the complexity of this transporter in the control of glycolysis. In contrast to liver cells where SLC13A5 is in the plasma membrane, the transporter is present almost exclusively in intracellular vesicles in presynaptic neuronal axons. This is critical to the control of cytoplasmic levels of citrate. If present in the plasma membrane, the transporter increases the levels of citrate in the cytoplasm; in contrast, if present in intracellular vesicles, the transporter sequesters citrate within these vesicles, thereby reducing the levels of citrate in the cytoplasm. This would have an opposing effect on glycolysis depending on whether the transporter is in the plasma membrane (inhibition of glycolysis) or in intracellular vesicles (activation of glycolysis). It has been speculated that the location and transport function of SLC13A5 in intracellular vesicles may be unique to excitable neurons because excitation-associated action potentials regulate trafficking of the transporter to these vesicles. This is an interesting and novel aspect of SLC13A5 regarding the control of cellular metabolism that might have significant impact on the role of this transporter in cancer cells. Recent evidence indicates that certain aggressive cancer cells can become excitable with action potentials and other electrical properties which were once deemed exclusive to neurons [101,102,103]. This phenomenon has been documented in glioblastoma cells and in small-cell lung cancer cells, and the excitability in these cells has been linked to proliferation, invasion, and metastasis. If a given cancer cell expresses SLC13A5, the subcellular localization of the transporter may depend on whether or not the cell harbors the feature of electrical excitation, which would subsequently decide whether glycolysis is inhibited or activated. This could have downstream effects on glutaminolysis and glutamine “addiction” because the inhibition of glycolysis may increase the dependence of the cancer cell on glutamine metabolism, whereas the stimulation of glycolysis may have the opposite effect.

In contrast to these acute effects of citrate on cancer cell metabolism, the long-term effects of citrate and citrate-derived itaconate include transcriptional changes and consequent metabolic reprogramming. Citrate- and itaconate-associated changes in the activities of AMPK, HIF-1α, HIF-2α, and mTOR fall into this category.

### 7.5. Resistance to Ferroptosis—Role of Citrate and Citrate-Derived Itaconate

Ferroptosis is a non-apoptotic cell death related to an excess amount of free labile iron in cells [104]. When the levels of free Fe^2+^ increase, it initiates a Fenton reaction in which hydrogen peroxide is converted into a hydroxyl radical. This highly reactive radical acts on polyunsaturated fatty acids present in membrane-associated phospholipids to generate lipid peroxy radicals by breaking the double bonds and consequently causes membrane damage. This is the primary mechanism underlying iron-induced ferroptosis. Even though the role of citrate in the control of ferroptosis has not been reported by direct experiments, the potential is there because of the various signaling pathways controlled by citrate. First, citrate is an iron chelator with the ability to bind free iron (both Fe^2+^ and Fe^3+^), which is expected to protect the cells from ferroptosis. Second, citrate oxidation in the citric acid cycle coupled to oxidative phosphorylation leads to suppression of AMPK activity (see above). AMPK has a complex relationship with ferroptosis depending on the cell context. Generally, AMPK inhibits ferroptosis because when AMPK is active, it suppresses fatty acid synthesis, thus decreasing the endogenous production of polyunsaturated fatty acids and protecting the cells from ferroptosis [105]. However, AMPK has also been shown to induce ferroptosis by inhibiting the cystine–glutamate exchanger SLC7A11 via phosphorylation of BECN1; the phosphorylated BECN1 binds to SLC7A11 and blocks its transport function [106]. The resultant decrease in cystine entry into cells decreases glutathione synthesis, leading to an inability of the cells to remove hydrogen peroxide and lipid peroxides. As such, suppression of AMPK by citrate may have opposing effects on ferroptosis in a cell-context-dependent manner. Third, citrate activates HIF-2α transcriptional activity by inhibiting asparaginyl hydroxylase (FIH), and HIF-2α signaling is an important regulator of iron homeostasis. The H^+^-coupled Fe^2+^ importer SLC11A2 (known as DMT1 or divalent metal transporter) is a key target for transcription by HIF-2α [107]. Therefore, citrate-mediated potentiation of HIF-2α signaling is likely to increase cellular levels of free iron, which could promote ferroptosis.

The situation becomes even more complex given the recent discovery of the citrate-derived metabolite itaconate and its profound effect on cellular antioxidant machinery and consequently on ferroptosis [108,109]. The relevance of itaconate to the current review is the finding that cellular citrate levels dictate itaconate production. The enzyme responsible for this is aconitate decarboxylase-1 (ACOD1), which is found mostly in the mitochondrial matrix, and its substrate for itaconate production is *cis*-aconitate, an intermediate in the conversion of citrate to isocitrate by aconitase-2. ACOD1 is upregulated in cancer [110]; the increased activity of ACOD1, coupled with increased levels of citrate, in cancer cells promotes the synthesis of itaconate. Recent studies have highlighted the importance of this metabolite not only in cancer biology but also in immunology [111,112]. Itaconate promotes cancer by enabling cancer cells to evade the immune system via the establishment of an immunosuppressive environment. As such, itaconate is now described as an oncometabolite as well as an immunometabolite. In the context of ferroptosis, itaconate, as a potent electrophile, covalently modifies the disulfide linkage in the NRF2-Keap1 complex and releases NRF2 in free form, thus facilitating its translocation into the nucleus and subsequent function as a transcription factor to potentiate the expression of genes related to cellular antioxidant machinery. The transcriptional targets of NRF2 include the cystine–glutamate exchanger SLC7A11 and the catalytic subunit of glutamate–cysteine ligase, the first enzyme in the synthesis of glutathione; together, these two gene products promote glutathione synthesis, thus leading to protection against ferroptosis.

Regarding the impact of citrate on ferroptosis, the complex role of citrate in iron homeostasis is important. As mentioned above, citrate is a chelating agent for iron (Fe^2+^ as well as Fe^3+^) inside the cells and in the extracellular milieu. In fact, any iron that is not bound to transferrin circulates in blood predominantly in the form of a citrate–iron complex. Citrate also regulates iron influx into cells via DMT1, mediated by citrate-dependent activation of HIF-2α. The increased expression of DMT1 is important not only for the cellular uptake of non-bound free Fe^2+^ but also for the entry of transferrin-bound iron, the most prevalent form of iron in circulation. The transferrin–Fe^3+^ is taken into normal cells as well as cancer cells by receptor-mediated endocytosis via the cell-surface transferrin receptor. In this process, the receptor-bound transferrin–Fe^3+^ ends up in lysosomes, where Fe^3+^ is liberated from transferrin due to the acidic pH within the lysosomes, and the released Fe^3+^ is then reduced to Fe^2+^ by the ferrireductase activity of STEAP3 [113]. The resultant Fe^2+^ is then exported out of the lysosomes by DMT1. The naturally occurring transmembrane H^+^ gradient across the lysosomal membrane provides the driving force for this export process because of the nature of DMT1 as an H^+^-coupled transporter.

There is also evidence for a reciprocal relationship between iron and citrate where the cellular status of iron regulates citrate metabolism. This mode of interaction occurs through the iron-dependent aconitases 1 and 2. Both the cytoplasmic aconitase-1 and the mitochondrial aconitase-2 contain Fe-S clusters that are obligatory for their catalytic activity [114]. Therefore, iron deficiency decreases the activity of these enzymes and hence suppresses the conversion of citrate into isocitrate. Consequently, under iron-deficient conditions, cellular levels of citrate increase, which then feeds into anabolic pathways, leading to increased synthesis of fatty acids and cholesterol [115]. In addition, cytoplasmic aconitase-1 is a bifunctional protein. When cellular iron levels are sufficient, the protein functions as an enzyme to convert citrate into isocitrate. However, when the cellular iron status is low, the Fe-S clusters are depleted in the protein, which leads to suppression of its function as an enzyme; instead, the protein with depleted Fe-S clusters assumes a different role as an iron-regulatory protein (IRP1) [116]. IRP1 binds to iron-responsive elements (IREs) in ferritin mRNA, transferrin receptor mRNA, and DMT1 mRNA and modulates their translation or stability. The binding in ferritin mRNA occurs at the 5′-untranslated region, which interferes with translation and reduces ferritin synthesis. In contrast, the binding to transferrin receptor mRNA and DMT1 mRNA is at the 3′-untranslated region which increases mRNA stability and hence facilitates the synthesis of transferrin receptor and DMT1. The decrease in ferritin and the increase in the transferrin receptor and DMT1 mediated by IRP1 (i.e., iron-depleted aconitase-1) together restore cellular levels of free iron.

Even though the dual functions of citrate as a ferroptosis blocker on the one hand and as a potential ferroptosis inducer on the other hand seem contradictory, this aspect has to be considered in the context of cancer cell metabolism. Cancer cells have high demands for iron to support their rapid proliferative rate and energy needs, and therefore they upregulate iron acquisition pathways. The citrate-FIH-HIF-2α axis may play an essential role in this context. While the increased iron entry may meet the enhanced iron demands in cancer cells, it also poses an enhanced risk for iron-dependent ferroptosis. Citrate as an iron chelator and citrate-derived itaconate as a potentiator of the NRF2-SLC7A11-glutathione axis to boost the cellular antioxidant machinery may perform an important task to mitigate the risk of ferroptotic cell death. These apparently opposing effects of citrate in ferroptosis are directly related to the unique conditions pertaining to the special metabolic context of cancer cells. Taken collectively, the role of citrate in iron homeostasis and ferroptosis in cancer cells is complex, and the net result might depend not only on the cellular levels of citrate and itaconate but also on the expression and functional status of various other inter-related components in a given cancer cell type that dictate the levels of labile iron and the status of the antioxidant machinery within the cell.

## 8. Tumor Microenvironment and SLC13A5-Mediated Citrate Uptake into Cancer Cells

One of the characteristic features of a tumor microenvironment in solid tumors is the acidic extracellular pH, which can be as low as 6.5 [117,118,119,120,121]. The primary mechanism underlying this acidic pH is the release of lactic acid by the tumor cells. Additional mechanisms contributing to this feature include the carbonic anhydrase CAIX, the Na^+^/H^+^ exchanger NHE1 (SLC9A1), and the V-type H^+^-ATPase. This acidic pH in the tumor microenvironment plays a critical role in tumor cell proliferation and metastasis. The acidic environment in solid tumors promotes tumor progression through several mechanisms, including the degradation of the extracellular matrix to facilitate tumor cell invasion, suppression of antitumor responses and establishment of an immunosuppressive microenvironment that enables the tumor cells to evade immune surveillance, promotion of tumor angiogenesis, and activation of intracellular signaling via proton-sensing G-protein-coupled receptors on tumor cells and stromal cells. Despite the generally accepted notion that the acidic tumor microenvironment promotes the growth and spread of tumor cells, the relevance of this acidic pH to the nutrition of tumor cells has received relatively less attention. We have recently highlighted this aspect of the tumor microenvironment [119]. There are several nutrient transporters whose transport function is activated by extracellular acidic pH, and the transported nutrient substrates via these transporters are obligatory for the optimal growth of tumor cells. This includes H^+^-coupled Fe^2+^ transporter DMT1 (SLC11A2), H^+^-coupled folate transporter SLC46A1, and H^+^-coupled peptide transporters SLC15A1 and SLC15A2. This list must also include the citrate transporter SLC13A5, which, though a Na^+^-dependent transporter, mediates H^+^ symport and hence exhibits much greater transport activity in the presence of an acidic extracellular pH. This feature might be particularly relevant to metastatic tumor cells that grow in bone because of the citrate-rich niche in the bone matrix along with the acidic microenvironment. Here, we emphasize the distinction between human SLC13A5 and rodent Slc13a5 because only the former is activated by extracellular acidic pH whereas the latter is not. The involvement of multiple driving forces (Na^+^ gradient, H^+^ gradient, and membrane potential) in the transport process mediated by human SLC13A5 is important for the concentrative accumulation of extracellular citrate within the cancer cells given the relatively low affinity (*K*_t_ = 0.7 mM) of the transporter for citrate. In most organs, cancer cells in primary tumors are exposed to low concentrations of extracellular citrate equivalent to the citrate levels in plasma (~0.15 mM). There are exceptions, however [4]. In the brain, astrocytes synthesize and secrete not only lactate and glutamine but also citrate for use in neuronal metabolism. Consequently, the levels of citrate in the brain’s extracellular milieu are much higher than in plasma (~0.4 mM). Within the bone matrix, citrate levels could reach millimolar levels during osteolysis because bone resorption during this process releases citrate in free form. Similarly, the prostate is also exceptional in terms of high levels of extracellular citrate, as prostate epithelial cells secrete massive amounts of citrate as the predominant organic component of prostatic fluid [27]. Notwithstanding this varying spectrum of extracellular citrate levels in different tissues under normal conditions, cancer cells at these sites may be exposed to higher concentrations of citrate than found under physiological conditions because cancer-associated stromal cells are known to secrete citrate [75]. The low affinity of human SLC13A5 coupled with the energization of its citrate uptake process by multiple driving forces ensure effective accumulation of citrate within cancer cells against a concentration gradient even under these conditions of a broad range of citrate concentrations in the extracellular milieu. These key differences in the transport mechanisms between human and rodent transporters regarding their affinities for citrate and their transport mechanisms involving cotransport with H^+^ ought to be taken into consideration while designing preclinical studies using laboratory animals in investigations of the functional relevance of SLC13A5 in bone metastasis. Humanized rodent models in which the endogenous Slc13a5 has been replaced with human SLC13A5 might be appropriate for such studies. The C57BL/6J-*Slc13a5^em1(SLC13A5/Lutzy^*/Mmjax mouse line, available from the Jackson Laboratory, may be suitable for these studies. This knockin/knockout mouse, which expresses human SLC13A5 in place of murine Slc13a5, was generated by inserting the human SLC13A5 cDNA into the mouse *Slc13a5* gene in such a way that the expression of the endogenous mouse Slc13a5 is disrupted while the human SLC13A5 is expressed instead, but still under the control of the mouse *Slc13a5* gene promoter.

## 9. Bone Metastasis: A Speculative Model for the Relevance of Citrate in the Bone Matrix

Several cancers metastasize to bone; this includes breast, prostate, lung, kidney, and pancreatic cancers. Among these, breast and prostate cancers standout because of the involvement of bone metastasis in about 75% of advanced cases of the disease. Often, bone metastasis in these cases represents the osteolytic type, meaning that the growth and spread of metastatic tumor cells lead to osteolysis (i.e., bone degradation) due to activation of osteoclasts. This has a significant impact on the tumor microenvironment. Osteoclasts secrete H^+^ and the resultant extracellular acidic pH is needed to degrade the bone matrix. As ~80% of the body’s citrate pool resides in the bone matrix, degradation of bone would lead to the release of citrate, thus creating a citrate-rich niche. As such, tumor cells that have metastasized to the bone would be exposed to a highly acidic microenvironment and elevated citrate concentrations. Although the seed-and-soil theory was proposed more than a century ago, the factors that make bone a preferred “soil” for the growth of metastatic cancer cells remain incompletely understood. It is likely that the unique metabolite-rich milieu in bone may not only facilitate the preferential seeding of cancer cells in the bone but also offer a fertile soil for them to grow.

The bone matrix under osteolytic conditions represents an environment likely to be rich in two metabolites, citrate and citrate-derived itaconate. This unique metabolite environment would not be found in any other organ. Cancer cells promote differentiation of bone-lysing osteoclasts [122]. Even though citrate-derived itaconate is recognized as an important signaling molecule in the modulation of immune function during inflammation and in cancer [18,19,20], osteoclasts also possess the enzymatic machinery to synthesize itaconate from citrate [123]. This is not surprising because macrophages and osteoclasts are biologically related as both cell types originate from a common precursor [124].

**Figure 3:**
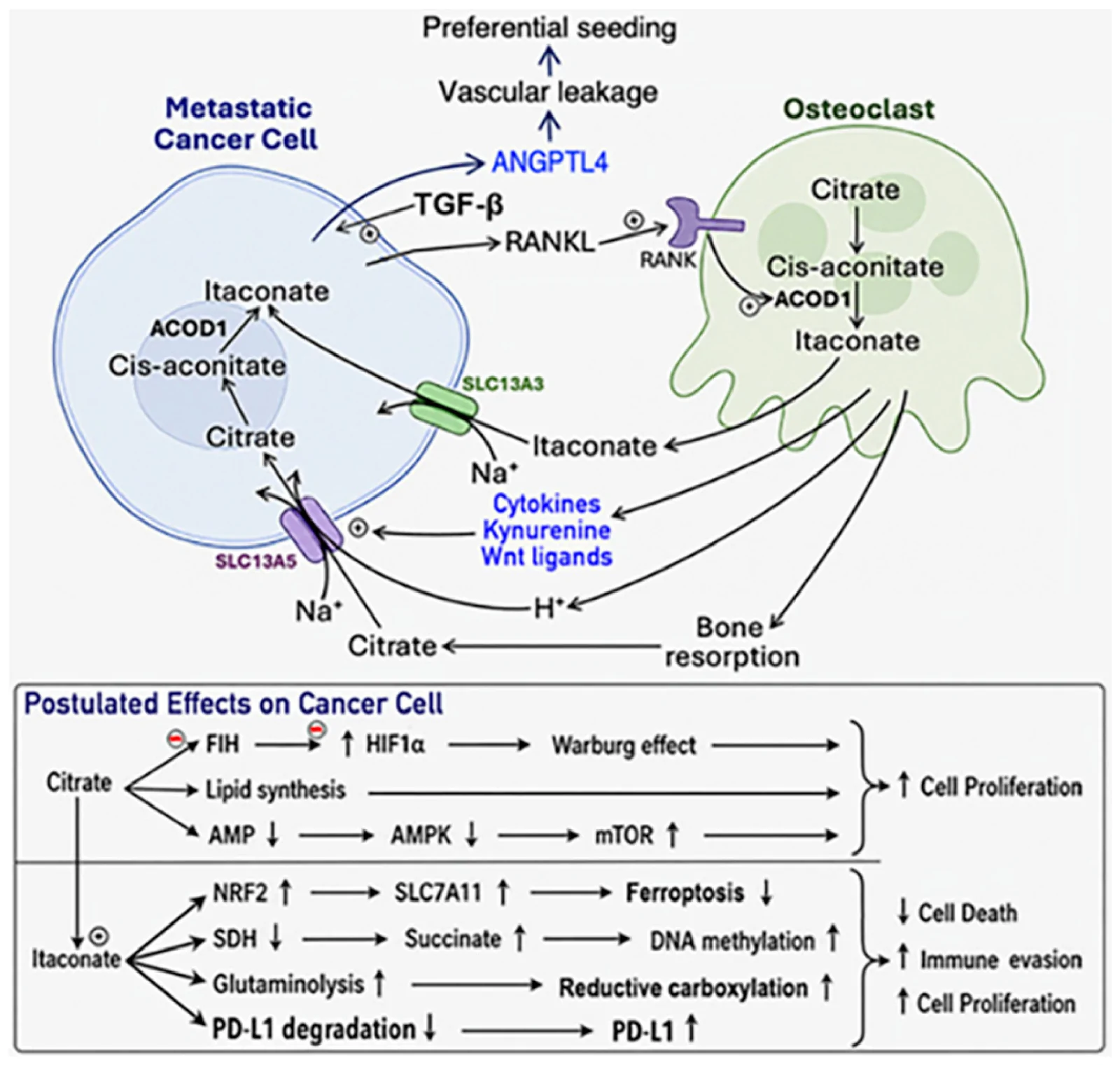
A speculative biochemical crosstalk between metastatic cancer cells and osteoclasts in the bone matrix. ANGPTL4, angiopoietin-like 4; TGF-β, transforming growth factor-β; ACOD1, aconitate decarboxylase-1; FIH, factor inhibiting HIF-1; HIF-1, hypoxia-inducible factor; AMPK, AMP-activated protein kinase; mTOR, mechanistic target of rapamycin; NRF2, nuclear factor erythroid 2-related factor 2; SDH, succinate dehydrogenase; PD-L1, programmed death ligand 1.

We propose a provocative, but experimentally testable, hypothesis to explain the seed-and-soil theory for bone metastasis (Figure 3). This hypothesis is based on potential active crosstalk between metastasized tumor cells and osteoclasts in the bone matrix. It has already been established that cancer cells induce osteoclast differentiation via the secretion of RANKL, a critical factor for the promotion of mononuclear bone marrow precursor cells into osteoclasts [122]. Breast cancer cells produce angiopoietin-like 4 to promote vascular leakage in situ and facilitate extravasation of cancer cells in the context of lung metastasis [125]; this is mediated by TGF-β in the tumor microenvironment. Interestingly, the bone matrix is the largest reservoir of TGF-β in the body [126]. Therefore, we hypothesize that the same phenomenon may also occur in the context of bone metastasis, where TGF-β induces secretion of angiopoietin-like 4 by cancer cells to promote vascular leakage and cancer cell extravasation in situ, thereby initiating the entry and seeding of cancer cells in bone matrix. The RANKL/RANK signaling which is promoted by the crosstalk between the cancer cells and osteoclasts may induce the enzymatic machinery to convert citrate into itaconate. Osteoclasts are also known to release H^+^ and accelerate bone lysis, which is likely to result in citrate release from the bone matrix. In addition, osteoclasts release Wnt ligands (activators of Wnt signaling), kynurenine (activator of the nuclear receptor AhR), and several proinflammatory cytokines (e.g., IL-6), which are all known to induce the transcription of the citrate transporter SLC13A5 [127]. As the extracellular milieu is rich in citrate, the induced SLC13A5 would mediate the entry of this citrate into cancer cells to promote anabolism and cell proliferation. The obligatory coupling of SLC13A5-mediated citrate transport to H^+^ symport dictates that this transport process is activated by the acidic tumor microenvironment that exists in the matrix, especially under the bone-lysing conditions.

Cancer cells also express the high-affinity dicarboxylate transporter SLC13A3 (also known as NaDC3) [128], which would facilitate the uptake of osteoclast-derived itaconate into cancer cells [129]. The resultant increase in cellular levels of citrate and itaconate may promote cancer cell proliferation, suppress ferroptotic cell death, and facilitate immune evasion. The biochemical pathways involved in these biological processes have been the subject of several recent reviews [18,19,20]. However, the schematic description of the biochemical mechanisms involved in the crosstalk between cancer cells and osteoclasts, as detailed in Figure 3, remains only speculative at present. Most of these pathways have been documented in other cell types, both normal and cancer cells. The working model in Figure 3 is based on this literature evidence generated in cellular contexts different from that of bone metastasis; therefore, direct experimentation is needed to validate the basic tenets of the proposed model in relation to bone metastasis. Furthermore, the proposed model may be applicable only to bone metastasis because the composition of the extracellular milieu in terms of metabolites and cytokines depicted in Figure 3 is dictated by the unique biology of the osteoclasts.

## 10. Therapeutic Implications and Future Directions

There is sufficient evidence for a tumor-promoting role for citrate and citrate-derived itaconate, not only in promoting cancer cell proliferation but also in facilitating immune evasion and resistance to cell death. In addition, given the obligatory role of citrate in the synthesis of fatty acids and cholesterol, citrate metabolism is currently considered a logical target for the treatment of hyperlipidemia, fatty liver, and diet-induced obesity. Most of the biological effects of citrate and itaconate are intracellular; therefore, the citrate transporters SLC13A5 and pmCIC, and the itaconate transporter SLC13A3, are essential for citrate and itaconate to elicit their tumor-promoting and fat-promoting functions. Some of the effects of citrate depend on the mitochondrial citrate transporter SLC25A1. The conversion of citrate into itaconate is obligatorily dependent on the activity of ACOD1. As such, pharmacological blockade of any of these proteins (SLC13A5, pmCIC, SLC25A1, SLC13A3 and ACOD1) has potential for cancer therapy and/or for enhancing the clinical efficacy of chemo/immunotherapies.

Several high-affinity inhibitors of SLC13A5 have been identified [130,131,132,133,134,135]. Some of these inhibitors have been shown to possess efficacy to block the transport function of SLC13A5 in vivo, but these studies have been done to address only the role of SLC13A5 in the liver as a promoter of fat synthesis and subsequent metabolic consequences such as diet-induced obesity, hyperlipidemia, and metabolic syndrome [134,135]. There appears to be no published report on any of these SLC13A5 inhibitors to assess the tumor-promoting role of SLC13A5 in cancer. The splice variant (pmCIC) of SLC25A1 which traffics to the plasma membrane to mediate the uptake of extracellular citrate into cancer cells has been investigated as a pharmacological target in cancer therapy using gluconate as a selective inhibitor of pmCIC [4,136]. Gluconate does not block the function of the mitochondrial variant SLC25A1. The efficacy of gluconate as an anti-cancer agent has been demonstrated in multiple cancer types in preclinical studies [4,29,75,77,136]. The mitochondrial SLC25A1 is inhibited by 1,2,3-benzene tricarboxylic acid (BTA) [137], 4-chloro-3-[[(3-nitrophenyl)amino]sulfonyl]-benzoic acid (CTPI-1) [138], its slightly modified variant CTPI-2 [23], and a new-generation inhibitor, CTPI-3 [139]. The efficacy of these inhibitors as anti-cancer agents has been validated in preclinical studies [23,68,74,139,140]. The pharmacokinetic properties of these inhibitors vary markedly. BTA exhibits low potency and low membrane permeability and, at the same time, lacks selectivity for SLC25A1. CTPI-1 has poor metabolic stability in vivo, thus necessitating high doses of the compound for therapeutic efficacy [138]. The newer derivatives, CTPI-2 and CTPI-3, not only exhibit higher potency to inhibit SLC25A1 than CTPI-1 but also possess better cell permeability and improved pharmacokinetics in vivo following oral administration [23,139]. In terms of metabolic stability and half-life in circulation, CTPI-3 is superior to CTPI-2, but both derivatives show desirable safety features. Despite these favorable pharmacokinetic and safety profiles, the studies of both derivatives are currently limited to preclinical animal models, and neither has advanced to clinical trials in humans.

SLC13A3 (also known as NaDC3) is a high-affinity Na^+^-coupled dicarboxylate transporter which recognizes various dicarboxylates (e.g., succinate) as its substrates [141]. It also functions as a transporter for the immunometabolite itaconate, and this transporter is inhibited by m-(methoxybenzyl)succinate (also known as SLC13A3i) [129]. The efficacy of this inhibitor in enhancing tumor immunity via resistance to ferroptotic cell death has been demonstrated [129]. The itaconate-producing enzyme ACOD1 is inhibited by citraconate [142], and the efficacy of this inhibitor to modulate inflammation, cell metabolism, and bacterial/viral infection by targeting ACOD1 has been validated [143,144].

Notwithstanding active research focusing on the development of drugs for the inhibition of transporters and enzymes related to citrate and citrate-derived itaconate, none of the candidates has yet advanced to clinical investigations in humans. The pharmaceutical company Pfizer developed PF-06649298 based on the hydroxysuccinic acid scaffold as an inhibitor of SLC13A5 [130,131], but there is no evidence of efforts to advance this compound further into clinical studies in humans, as deduced from peer-reviewed publications or from the company’s official clinical pipeline reports. Two new compounds, one containing the hydroxysuccinic acid scaffold similar to that in PF-06649298 [135] and the other (BI01383298) structurally unrelated to PF-06649298 [132,133], have been shown to inhibit SLC13A5 with nanomolar potency. However, both compounds remain in the early stages of development, with studies thus far limited to in vitro experiments or preclinical evaluation in animal models. The BI01383298 compound is species-selective; it inhibits only human SLC13A5 and has no effect on mouse Slc13a5 [132,133]. Even though it possesses nanomolar affinity to inhibit human SLC13A5, no attempts have been made to determine its pharmacokinetic properties in laboratory animal models because of this restrictive species selectivity. Nonetheless, it is a very useful inhibitor for cell-based in vitro experiments involving human SLC13A5. In contrast to BI01383298, the PF-06649298 compound inhibits both mouse and human SLC13A5s, is orally active and metabolically stable in circulation, and exhibits favorable pharmacokinetic features and the ability to inhibit the function of the transporter in the liver in vivo [130,131]. The newer derivative of PF-06649298, designated LBA-3, shows significant improvement over the parent compound in pharmacokinetic properties and oral bioavailability and also has no detectable systemic toxicity [135]. Nonetheless, as indicated earlier, none of these inhibitors has yet advanced beyond preclinical animal studies. Several hurdles remain in advancing SLC13A5 inhibitors to clinical trials, including optimization of their pharmacokinetic and pharmacodynamic properties and determination of the most appropriate route of administration (oral or injectable). An even more serious concern is the potential for side effects. Loss-of-function mutations in SLC13A5 cause a devastating disease in humans, highlighting the essential role of SLC13A5 in brain development and neurological functions. As such, any potentially useful SLC13A5 inhibitor for the treatment of cancer or fatty liver/hyperlipidemia must have no or limited ability to cross the blood–brain barrier [45]. This puts significant restrictions on successful development of SLC13A5 inhibitors for therapeutic purposes in cancer and liver diseases (e.g., nonalcoholic fatty liver disease, also known as metabolic dysfunction-associated steatotic liver disease). However, the neurological disease associated with SLC13A5 deficiency (EIEE-25) is congenital, and it is not known whether pharmacological suppression of the transport function of SLC13A5 later in life as a strategy to treat cancer or other diseases would have detrimental effects on brain function similar to what is seen in EIEE-25, which results from congenital absence of the transporter function. There is also an option to design and develop SLC13A5 inhibitors that do not cross the blood–brain barrier and thus do not have significant negative effects on brain function. Such inhibitors could have potential in the treatment of cancer and liver diseases that occur outside the brain. SLC25A1 inhibitors face a different kind of challenge. Unlike SLC13A5, which is expressed only in selective tissues, SLC25A1 is expressed ubiquitously. Therefore, pharmacological blockade of this widely expressed transporter for any given therapeutic purpose may exhibit an increased risk of unwanted side effects.

Interest in ACOD1 as a drug target has been on the rise in recent years because of the discovery of itaconate not only as an immunometabolite but also as an oncometabolite. The expression of ACOD1 is restricted only to immune cells (macrophages and neutrophils), osteoclasts, and tumor cells. As itaconate promotes an immunosuppressive environment in the context of cancer, blockade of its production via the inhibition of ACOD1 has the potential to boost antitumor immunity and hence to enhance the clinical efficacy of cancer immunotherapy. There is evidence of active ongoing research in ACOD1 inhibitors as potential therapeutic agents; however, to date, no ACOD1 inhibitor has yet been validated for clinical use in humans.

Despite the lack of success in advancing any of the inhibitors of citrate transporters and citrate metabolism into clinical trials, it is likely that continued efforts in this area will eventually come to fruition in developing novel therapeutic agents targeting SLC13A5 and/or ACOD1 for the treatment of fatty liver and cancer.

## Data Availability

No new data were created or analyzed in this study. Data sharing is not applicable to this article.

## References

[1] Krebs H.A., Johnson W.A. (1980). The role of citric acid in intermediate metabolism in animal tissues. FEBS Lett..

[2] Batchuluun B., Pinkosky S.L., Steinberg G.R. (2022). Lipogenesis inhibitors: Therapeutic opportunities and challenges. Nat. Rev. Drug Discov..

[3] Cui D., Yu X., Guan Q., Shen Y., Liao J., Liu Y., Su Z. (2025). Cholesterol metabolism: Molecular mechanisms, biological functions, diseases, and therapeutic targets. Mol. Biomed..

[4] Parkinson E.K., Adamski J., Zahn G., Gaumann A., Flores-Borja F., Ziegler C., Mycielska M.E. (2021). Extracellular citrate and metabolic adaptations of cancer cells. Cancer Metastasis Rev..

[5] Mosaoa R., Kasprzyk-Pawelec A., Fernandez H.R., Avantaggiati M.L. (2021). The mitochondrial citrate carrier SLC25A1/CIC and the fundamental role of citrate in cancer, inflammation and beyond. Biomolecules.

[6] Pajor A.M. (2014). Sodium-coupled dicarboxylate and citrate transporters from the SLC13 family. Pflug. Arch..

[7] Jaramillo-Martinez V., Urbatsch I.L., Ganapathy V. (2021). Functional distinction between human and mouse sodium-coupled citrate transporters and its biological significance: An attempt for structural basis using a homology modeling approach. Chem. Rev..

[8] Mimura Y., Yasujima T., Inoue K., Akino S., Namba C., Kusuhara H., Sekiguchi Y., Ohta K., Yamashiro T., Yuasa H. (2024). SLC35G1 is a highly chloride-sensitive transporter responsible for the basolateral membrane transport in intestinal citrate absorption. eLife.

[9] Szeri F., Lundkvist S., Donnelly S., Engelke U.F.H., Rhee K., Williams C.J., Sundberg J.P., Wevers R.A., Tomlinson R.E., Jansen R.S. (2020). The membrane protein ANKH is crucial for bone mechanical performance by mediating cellular export of citrate and ATP. PLoS Genet..

[10] Schwertner B., Dahdal G., Jagla W., Grossmann L., Drexler K., Krahn M.P., Evert K., Berneburg M., Haferkamp S., Ziegler C. (2024). Expression of the plasma membrane citrate carrier (pmCIC) in human cancerous tissues—Correlation with tumour aggressiveness. Front. Cell Dev. Biol..

[11] Davies E., Muller K.H., Wong W.C., Pickard C.J., Reid D.G., Skepper J.N., Duer M.J. (2014). Citrate bridges between mineral platelets in bone. Proc. Natl. Acad. Sci. USA.

[12] Ogura J., Sato T., Higuchi K., Sivaprakasam S., Kopel J., Bhutia Y.D., Ganapathy V. (2022). Binding of citrate-Fe^3+^ to plastic culture dishes, an artefact useful as a simple technique to screen for new iron chelators. Int. J. Mol. Sci..

[13] Wellen K.E., Hatzivassiliou G., Sachdeva U.M., Bui T.V., Cross J.R., Thompson C.B. (2009). ATP-citrate lyase links cellular metabolism to histone acetylation. Science.

[14] Pouikli A., Maleszewska M., Parekh S., Yang M., Nikopoulou C., Bonfiglio J.J., Mylonas C., Sandoval T., Schumacher A.L., Hinze Y. (2022). Hypoxia promotes osteogenesis by facilitating acetyl-CoA-mediated mitochondrial-nuclear communication. EMBO J..

[15] Dieterich I.A., Cui Y., Braun M.M., Lawton A.J., Robinson N.H., Peotter J.L., Yu Q., Casler J.C., Glick B.S., Audhya A. (2021). Acetyl-CoA flux from the cytosol to the ER regulates engagement and quality of the secretory pathway. Sci. Rep..

[16] McGregor E.R., McGill C.J., Arp N.L., Clark J.P., Baldwin D.A., Kuang D., Fernandez-Fuente G., Choi Y.H., Pearson K.S., Nagorski J.K. (2026). Acetyl-CoA availability regulates neuronal metabolism, growth, and synaptic activity. bioRxiv.

[17] Franklin R.B., Chellaiah M., Zou J., Reynolds M.A., Costello L.C. (2014). Evidence that osteoblasts are specialized citrate-producing cells that provide the citrate for incorporation into the structure of bone. Open Bone J..

[18] Williams N.C., O’Neill L.A.J. (2018). A role for the Krebs cycle intermediate citrate in metabolic reprogramming in innate immunity and inflammation. Front. Immunol..

[19] Haase L., Frezza C. (2024). Itaconate promotes an unexpected tumor immune escape mechanism. Cancer Cell.

[20] Peace C.G., O’Neill L.A. (2022). The role of itaconate in hose defense and inflammation. J. Clin. Investig..

[21] Elkhaled A., Jalbert L.E., Phillips J.J., Yoshihara H.A., Parvataneni R., Srinivasan R., Bourne G., Berger M.S., Chang S.M., Cha S. (2012). Magnetic resonance of 2-hydroxyglutarate in IDH1-mutated low-grade gliomas. Sci. Transl. Med..

[22] Bisaccia F., De Palma A., Dierks T., Kramer R., Palmieri F. (1993). Reaction mechanism of the reconstituted tricarboxylate carrier from rat liver mitochondria. Biochim. Biophys. Acta.

[23] Fernandez H.R., Gadre S.M., Tan M., Graham G.T., Mosaoa R., Ongkeko M.S., Kim K.A., Riggins R.B., Parasido E., Petrini I. (2018). The mitochondrial citrate carrier, SLC25A1, drives stemness and therapy resistance in on-small cell lung cancer. Cell Death Diff..

[24] Kaplan R.S., Mayor J.A., Wood D.O. (1993). The mitochondrial tricarboxylate transport protein. cDNA cloning, primary structure, and comparison with other mitochondrial transport proteins. J. Biol. Chem..

[25] Goldmuntz E., Wang Z., Roe B.A., Budarf M.L. (1996). Cloning, genomic organization, and chromosomal localization of human citrate transport protein to the DiGeorge/velocardiofacial syndrome minimal critical region. Genomics.

[26] Iacobazzi V., Lauria G., Palmieri F. (1997). Organization and sequence of the human gene for the mitochondrial citrate transport protein. DNA Seq..

[27] Mazurek M.P., Prasad P.D., Gopal E., Fraser S.P., Bolt L., Rizaner N., Palmer C.P., Foster C.S., Palmieri F., Ganapathy V. (2010). Molecular origin of plasma membrane citrate transporter in human prostate epithelial cells. EMBO Rep..

[28] Mycielska M.E., Djamgoz M.B. (2004). Citrate transport in the human prostate epithelial PNT2-C2 cell line: Electrophysiological analyses. J. Physiol..

[29] Mycielska M.E., Dettmer K., Rummele P., Schmidt K., Prehn C., Milenkovic V.M., Jagle W., Madej G.M., Lanlow M., Schladt M. (2018). Extracellular citrate affects critical elements of cancer cell metabolism and supports cancer development in vivo. Cancer Res..

[30] Mycielska M.E., Palmer C.P., Brackenbury W.J., Djamgoz M.B.A. (2005). Expression of Na^+^-dependent citrate transport in a strongly metastatic human prostate cancer PC-3M cell line: Regulation by voltage-gated Na^+^ channel activity. J. Physiol..

[31] Inoue K., Zhuang L., Maddox D.M., Smith S.B., Ganapathy V. (2002). Structure, function, and expression pattern of a novel sodium-coupled transporter (NaCT) cloned from mammalian brain. J. Biol. Chem..

[32] Inoue K., Zhuang L., Ganapathy V. (2002). Human Na^+^-coupled citrate transporter: Primary structure, genomic organization, and transport function. Biochem. Biophys. Res. Commun..

[33] Inoue K., Fei Y.J., Zhuang L., Gopal E., Miyauchi S., Ganapathy V. (2004). Functional features and genomic organization of mouse NaCT, a sodium-coupled transporter for tricarboxylic acid cycle intermediates. Biochem. J..

[34] Gopal E., Miyauchi S., Martin P.M., Ananth S., Srinivas S.R., Smith S.B., Prasad P.D., Ganapathy V. (2007). Expression and functional features of NaCT, a sodium-coupled citrate transporter, in human and rat livers and cell lines. Am. J. Physiol. Gastrointest. Liver Physiol..

[35] Inoue K., Zhuang L., Maddox D.M., Smith S.B., Ganapathy V. (2003). Human sodium-coupled citrate transporter, the orthologue of Drosophila Indy, as a novel target for lithium action. Biochem. J..

[36] Gopal E., Babu E., Ramachandran S., Bhutia Y.D., Prasad P.D., Ganapathy V. (2015). Species-specific influence of lithium on the activity of SLC13A5 (NaCT): Lithium-induced activation is specific for the transporter in primates. J. Pharmacol. Exp. Ther..

[37] Pajor A.M. (1995). Sequence and functional characterization of a renal sodium/dicarboxylate cotransporter. J. Biol. Chem..

[38] Pajor A.M. (1996). Molecular cloning and functional expression of a sodium-dicarboxylate cotransporter from human kidney. Am. J. Physiol..

[39] Sekine T., Cha S.H., Hosoyamada M., Kanai Y., Watanabe N., Furuta Y., Fukuda K., Igarashi T., Endou H. (1998). Cloning, functional characterization, and localization of a rat renal Na^+^-dicarboxylate transporter. Am. J. Physiol..

[40] Pajor A.M., Sun N.N. (2000). Molecular cloning, chromosomal organization, and functional characterization of a sodium-dicarboxylate cotransporter from mouse kidney. Am. J. Physiol. Ren. Physiol..

[41] Pajor A.M. (1999). Citrate transport by the kidney and intestine. Semin. Nephrol..

[42] Zomorodian A., Moe O.W. (2025). Citrate and calcium kidney stones. Clin. Kidney.

[43] Wathuliyadde N., Willmore K.E., Kelly G.M. (2026). Molecular mechanisms of ANKH and regulation in skeletal mineralization. JBMR Plus.

[44] Williams C.J. (2016). The role of ANKH in pathologic mineralization of cartilage. Curr. Opin. Rheumatol..

[45] Kopel J.J., Bhutia Y.D., Sivaprakasam S., Ganapathy V. (2021). Consequences of NaCT/SLC13A5/mINDY deficiency: Good versus evil, separated only the blood-brain barrier. Biochem. J..

[46] Pop A., Williams M., Struys E.A., Monne M., Jansen E.E., Grassi A.D., Kanhai W.A., Scarcia P., Fernandez Ojeda M.R., Porcelli V. (2018). An overview of combined D-2 and L-2-hydroxyglutaric aciduria: Functional analysis of CIC variants. J. Inherit. Metab. Dis..

[47] Chaouch A., Porcelli V., Cox D., Edvardson S., Scarcia P., De Grassi A., Pierri C.L., Cossins J., Laval S.H., Griffin H. (2014). Mutations in the mitochondrial citrate carrier SLC25A1 are associated with impaired neuromuscular transmission. J. Neuromuscul. Dis..

[48] Ye D., Guan K.L., Xiong Y. (2018). Metabolism, activity, and targeting of D- and L-hydroxyglutarates. Trends Cancer.

[49] Muhlhausen C., Salomons G.S., Lukacs Z., Struys E.A., van der Knaap M.S., Ullrich K., Santer R. (2014). Combined D2-/L2-hydroxyglutaric aciduria (SLC25A1 deficiency): Clinical course and effects of citrate treatment. J. Inherit. Metab. Dis..

[50] Alzahrani A.Y.B., Alghamdi L.S.A., Alghamdi H.A.M., Hassan A.F., Alsehemi M.A. (2023). Congenital myasthenic syndrome associated with SLC25A1 gene variant: The first reported case in Saudi Arabia. Cureus.

[51] Aruga S., Wehrli S., Kaissling B., Moe O.W., Preisig P., Pajor A.M., Alpern R.J. (2000). Chronic metabolic acidosis increases NaDC-1 mRNA and protein abundance in rat kidney. Kidney Int..

[52] Yang X., Yao S., An J., Jin H., Wang H., Tuo B. (2021). SLC26A6 and NaDC1: Future direction of nephrolithiasis and calculus-related hypertension research. Mol. Med. Rep..

[53] http://www.tessresearch.org/understanding-slc13a5/.

[54] Brown T.L., Bainbridge M.N., Zahn G., Nye K.L., Porter B.E. (2024). The growing research toolbox for SLC13A5 citrate transporter disorder: A rare disease with animal models, cell lines, an ongoing natural history study and an engaged patient advocacy organization. Ther. Adv. Rare Dis..

[55] Jaramillo-Martinez V., Ganapathy V., Urbatsch I.L. (2021). A home run for human NaCT/SLC13A5/INDY: Cryo-EM structure and homology model to predict transport mechanisms, inhibitor interactions and mutational defects. Biochem. J..

[56] Jaramillo-Martinez V., Sennoune S.R., Tikhonova E.B., Karamyshev A.L., Ganapathy V., Urbatsch I.L. (2024). Molecular phenotypes segregate missense mutations in SLC13A5 epilepsy. J. Mol. Biol..

[57] Willmes D.M., Kurzbach A., Henke C., Schumann T., Zahn G., Heifetz A., Jordan J., Helfand S.L., Birkenfeld A.L. (2018). The longevity gene INDY (I’m Not Dead Yet) in metabolic control: Potential as pharmacologic target. Pharmacol. Ther..

[58] Mishra D., Kannan K., Meadows K., Macro J., Li M., Frankel S., Rogina B. (2021). INDY—From flies to worms, mice, rats, non-human primates, and humans. Front. Aging.

[59] Bhutia Y.D., Kopel J.J., Lawrences J.J., Neugebauer V., Ganapathy V. (2017). Plasma membrane Na^+^-coupled citrate transporter (SLC13A5) and neonatal epileptic encephalopathy. Molecules.

[60] Jaramillo-Martinez V., Sivaprakasam S., Ganapathy V., Urbatsch I.L. (2021). Drosophila INDY and mammalian INDY: Major differences in transport mechanism and structural features despite mostly similar biological functions. Metabolites.

[61] Kumar A., Cordes T., Thalacker-Mercer A.E., Pajor A.M., Murphy A.N., Metallo C.M. (2021). NaCT/SLC13A5 facilitates citrate import and metabolism under nutrient-limited conditions. Cell Rep..

[62] Fernandez-Fuente G., Overmyer K.A., Lawton A.J., Kasza I., Shapiro S.L., Gallego-Munoz P., Coon J.J., Denu J.M., Alexander C.M., Puglielli L. (2023). The citrate transporters SLC13A5 and SLC25A1 elicit different metabolic responses and phenotypes in the mouse. Commun. Biol..

[63] Fernandez-Fuente G., Rigby M.L., Puglielli L. (2023). Intracellular citrate/acetyl-CoA flux and endoplasmic reticulum acetylation: Connectivity is the answer. Mol. Metab..

[64] Zhang Y., Shen X., Shen Y., Wang C., Yu C., Han J., Cao S., Qian L., Ma M., Huang S. (2026). Cytosolic acetyl-coenzyme A is a signalling metabolite to control mitophagy. Nature.

[65] Koshenov Z., Kokotos A.C., Benedetti L., Schwartz J.L., Ryan T.A. (2025). A genetic driver of epileptic encephalopathy impairs gating of synaptic glycolysis. bioRxiv.

[66] https://ualcan.path.uab.edu/cgi-bin/TCGAExResultNew2.pl?genenam=SLC25A1&ctype=THCA.

[67] You X., Huang L., Huang O., Deng Y., Shi X. (2023). A comprehensive analysis of SLC25A1 expression and its oncogenic role in pan-cancer. Discov. Oncol..

[68] Kolukula V.K., Sahu G., Wellstein A., Rodriguez O.C., Preet A., Iacobazzi V., D’Orazi G., Albanese C., Palmieri F., Avantaggiati M.L. (2014). SLC25A1, or CIC, is a novel transcriptional target of mutant p53 and a negative tumor prognostic marker. Oncotarget.

[69] Catalina-Rodriguez O., Kolukula V.K., Tomita Y., Preet A., Palmieri F., Wellstein A., Byers S., Giaccia A.J., Glasgow E., Albanese C. (2012). The mitochondrial citrate transporter, CIC, is essential for mitochondrial homeostasis. Oncotarget.

[70] Ozkaya A.B., Ak H., Atay S., Aydin H.H. (2015). Targeting mitochondrial citrate transport in breast cancer cell lines. Anticancer Agents Med. Chem..

[71] Bhalla K., Hwang B.J., Dewi R.E., Ou L., Twaddel W., Fang H.B., Vafai S.B., Vazquez F., Puigserver P., Boros L. (2011). PGC1α promotes tumor growth by inducing gene expression programs supporting lipogenesis. Cancer Res..

[72] Zhang Z., Qiao Y., Sun Q., Peng L., Sun L. (2023). A novel SLC25A1 inhibitor, parthenolide, suppresses the growth and stemness of liver cancer stem cells with metabolic vulnerability. Cell Death Discov..

[73] Shahid M., Ashfaq I., Haider B., Tayyeb A. (2026). SLC25A1 drives lipid metabolic reprogramming promoting liver injury leading to hepatocellular carcinoma. Mol. Biol. Rep..

[74] Hlouschek J., Hansel C., Jendrossek V., Matschke J. (2018). The mitochondrial citrate carrier (SLC25A1) sustains redox homeostasis and mitochondrial metabolism supporting radioresistance of cancer cells with tolerance to cycling severe hypoxia. Front. Oncol..

[75] Drexler K., Schmidt K.M., Jordan K., Federlin M., Milenkovic V.M., Liebisch G., Artati A., Schmidl C., Madej G., Tokarz J. (2021). Cancer-associated cells release citrate to support tumour metastatic progression. Life Sci. Alliance.

[76] Drexler K., Schwertner B., Haerteis S., Aung T., Berneburg M., Geissler E.K., Mycielska M.E., Haferkamp S. (2022). The role of citrate homeostasis in Merkel cell carcinoma pathogenesis. Cancers.

[77] Drexler K., Schwertner B., Zenderowski V., Schreieder L., Harrer D.C., Berneburg M., Geissler E., Mycielska M., Haferkamp S. (2026). The influence of extracellular citrate in physiological concentration on the proliferation of malignant melanoma. J. Cell Mol. Med..

[78] http://ualcan.path.uab.edu/cgi-bin/TCGAExResultNew2.pl?genenam=SLC13A5&ctype=PAA.

[79] Li Z., Li D., Choi E.Y., Lapidus R., Zhang L., Huang S.M., Shapiro P., Wang H. (2017). Silencing of solute carrier family 13 membrane 5 disrupts energy homeostasis and inhibits proliferation of human hepatocarcinoma cells. J. Biol. Chem..

[80] http://ualcan.path.uab.edu/cgi-bin/TCGAExResultNew2.pl?genenam=SLC13A5&ctype=LIHC.

[81] Bhutia Y.D., Babu E., Ramachandran S., Ganapathy V. (2015). Amino acid transporters in cancer and their relevance to “glutamine addiction”: Novel targets for the design of a new class of anticancer drugs. Cancer Res..

[82] Mathew M., Nguyen N.T., Bhutia Y.D., Sivaprakasam S., Ganapathy V. (2024). Metabolic signature of Warburg effect in cancer: An effective and obligatory interplay between nutrient transporters and catabolic/anabolic pathways to promote tumor growth. Cancers.

[83] Bhutia Y.D., Ganapathy V. (2016). Glutamine transporters in mammalian cells and their functions in physiology and cancer. Biochim. Biophys. Acta.

[84] Sniegowski T., Korac K., Bhutia Y.D., Ganapathy V. (2021). SLC6A14 and SLC38A5 drive the glutaminolysis and serine-glycine-one-carbon pathways in cancer. Pharmaceuticals.

[85] Fendt S.M., Bell E.L., Keibler M.A., Olenchock B.A., Mayers J.R., Wasylenko T.M., Vokes N.I., Guarente L., Vander Heiden M.G., Stephanopoulos G. (2013). Reductive glutamine metabolism is a function of the α-ketoglutarate to citrate ratio in cells. Nat. Commun..

[86] Mullen A.R., Hu Z., Shi X., Jiang L., Boroughs L.K., Kovacs Z., Boriack R., Rakheja D., Sullivan L.B., Linehan W.M. (2014). Oxidation of α-ketoglutarate is required for reductive carboxylation in cancer cells with mitochondrial defects. Cell Rep..

[87] Inoki K., Kim J., Guan K.L. (2012). AMPK and mTOR in cellular energy homeostasis and drug targets. Annu. Rev. Pharmacol. Toxicol..

[88] Peters J.M. (2017). Flipping a citrate switch on liver cancer cells. J. Biol. Chem..

[89] Dodd K.M., Yang J., Shen M.H., Sampson J.R., Tee A.R. (2014). mTORC1 drives HIF-1α and VEGF-A signaling via multiple mechanisms involving 4E-BP1, S6K1 and STAT3. Oncogene.

[90] Land S.C., Tee A.R. (2007). Hypoxia-inducible factor 1α is regulated by the mammalian target of rapamycin (mTOR) via an mTOR signaling motif. J. Biol. Chem..

[91] Koivunen P., Hirsila M., Remes A.M., Hassinen I.E., Kivirikko K.I., Myllyharju J. (2007). Inhibition of hypoxia-inducible factor (HIF) hydroxylases by citric acid cycle intermediates: Possible links between cell metabolism and stabilization of HIF. J. Biol. Chem..

[92] Hewitson K.S., Lienard B.M., McDonough M.A., Clifton I.J., Butler D., Soares A.S., Oldham N.J., McNeill L.A., Schofield C.J. (2007). Structural and mechanistic studies on the inhibition of the hypoxia-inducible transcription factor hydroxylases by tricarboxylic acid cycle intermediates. J. Biol. Chem..

[93] Murugesan T., Rajajeyabalachandran G., Kumar S., Nagaraju S., Jegatheesan S.K. (2018). Targeting HIF-2α as therapy for advanced cancers. Drug Discov. Today.

[94] Bakleh M.Z., Zen A.A.H. (2025). The distinct role of HIF-1α and HIF-2α in hypoxia and angiogenesis. Cells.

[95] Keith B., Johnson R.S., Simon M.C. (2011). HIF-1α and HIF-2α: Sibling rivalry in hypoxic tumor growth and progression. Nat. Rev. Cancer.

[96] Xu R., Wang K., Rizzi J.P., Huang H., Grina J.A., Schlachter S.T., Wang B., When P.M., Yang H., Dixon D.D. (2019). 3-[(1*S*,2*S*,3*R*)-2,3-Difluoro-1-hydroxy-7-methylsulfonylindan-4-yl]oxy-5-fluorobenzonitrile (PT2977), a hypoxia-inducible factor 2α (HIF-2α) inhibitor for the treatment of clear cell renal cell carcinoma. J. Med. Chem..

[97] Sim J., Cowburn A.S., Palazon A., Madhu B., Tyrakis P.A., Macias D., Bargiela D.M., Pietsch S., Gralla M., Evans C.E. (2018). The factor inhibiting HIF asparaginyl hydroxylase regulates oxidative metabolism and accelerates metabolic adaptation to hypoxia. Cell Metab..

[98] DeBerardinis R.J., Mancuso A., Daikhin E., Nissim I., Yudkoff M., Wehrli S., Thompson C.B. (2007). Beyond aerobic glycolysis: Transformed cells can engage in glutamine metabolism that exceeds the requirement for protein and nucleotide synthesis. Proc. Natl. Acad. Sci. USA.

[99] Damiani C., Colombo R., Gaglio D., Mastroianni F., Pescini D., Westerhoff H.V., Mauri G., Vanoni M., Alberghina L. (2017). A metabolic core model elucidates how enhanced utilization of glucose and glutamine, with enhanced glutamine-dependent lactate production, promotes cancer cell growth: The WarburQ effect. PLoS Comput. Biol..

[100] Rajagopalan K.N., DeBerardinis R.J. (2011). Role of glutamine in cancer: Therapeutic and imaging implications. J. Nucl. Med..

[101] Peinado P., Stazi M., Ballabio C., Margineanu M.B., Li Z., Colon C.I., Hsieh M.S., Choudhuri S.P., Stastny V., Hamilton S. (2025). Intrinsic electrical activity drives small-cell lung cancer progression. Nature.

[102] Tong T., Hendriksen J.D., Elbaek K.L., Ozsvar A., Eschen J.T., Rodriguez-Gonzalez F.G., Pusat I.D., Christensen E.L., Rahbaek M., Frederiksen K.P. (2024). Glioblastoma cells imitate neuronal excitability in humans. bioRxiv.

[103] Djamgoz M.B.A. (2024). Electrical excitability of cancer cells—CELEX model updated. Cancer Metastasis Rev..

[104] Stockwell B.R. (2022). Ferroptosis turns 10: Emerging mechanisms, physiological functions, and therapeutic applications. Cell.

[105] Lee H., Zandkarimi F., Zhang Y., Meena J.K., Kim J., Zhuang L., Tyagi S., Ma L., Westbrook T.F., Steinberg G.R. (2020). Energy-stress-mediated AMPK activation inhibits ferroptosis. Nat. Cell Biol..

[106] Song X., Zhu S., Chen P., Hou W., Wen Q., Liu J., Xie Y., Liu J., Klionsky D.J., Kroemer G. (2018). AMPK-mediated BECN1 phosphorylation promotes ferroptosis by directly blocking system x_c_^-^ activity. Curr. Biol..

[107] Xue X., Taylor M., Anderson E., Hao C., Qu A., Greenson J.K., Zimmermann E.M., Gonzalez F.J., Shah Y.M. (2012). Hypoxia-inducible factor-2α activation promotes colorectal cancer progression by dysregulating iron homeostasis. Cancer Res..

[108] McGettrick A.F., Bourner L.A., Dorsey F.C., O’Neill L.A.J. (2024). Metabolic messengers: Itaconate. Nat. Metab..

[109] Jimenez-Canete N., Aliena-Valero A., Bressan C.A., Perez S., Torres-Cuevas I., Benkenstein L., Pinto S., Cuadrado A., Sastre J., Rius-Perez S. (2026). Obesity suppresses neutrophil-dependent itaconate signaling promoting ferroptosis in acute pancreatitis. Redox Biol..

[110] Wang Z., Cui L., Lin Y., Huo B., Zhang H., Xie C., Zhang H., Liu Y., Jin H., Guo H. (2024). Cancer cell-intrinsic biosynthesis of itaconate promotes tumor immunogenicity. EMBO J..

[111] Li R., Zhang P., Wang Y., Tao K. (2020). Itaconate: A metabolite regulates inflammation response and oxidative stress. Oxid. Med. Cell. Longev..

[112] O’Neill L.A., Artyomov M.N. (2019). Itaconate: The poster child of metabolic reprogramming in macrophage function. Nat. Rev. Immunol..

[113] Knutson M.D. (2007). Steap proteins: Implications for iron and copper metabolism. Nutr. Rev..

[114] Tong W.H., Rouault T.A. (2007). Metabolic regulation of citrate and iron by aconitases: Role of iron-sulfur cluster biogenesis. Biometals.

[115] Pereira M., Chen T.D., Buang N., Olona A., Ko J.H., Prendecki M., Costa A.S.H., Nikitopoulou E., Tronci L., Pusey C.D. (2019). Acute iron deprivation reprograms human macrophage metabolism and reduces inflammation in vivo. Cell Rep..

[116] Rouault T.A., Maio N. (2017). Biogenesis and functions of mammalian iron-sulfur proteins in the regulation of iron homeostasis and pivotal metabolic pathways. J. Biol. Chem..

[117] Justus C.R., Dong L., Yang L.V. (2013). Acidic tumor microenvironment and pH-sensing G-protein-coupled receptors. Front. Physiol..

[118] Hosonuma M., Yoshimura K. (2023). Association between pH regulation of the tumor microenvironment and immunological state. Front. Oncol..

[119] Brown T.L., Ganapathy V. (2020). Lactate/GPR81 signaling and proton motive force in cancer: Role in angiogenesis, immune escape, nutrition, and Warburg phenomenon. Pharmacol. Ther..

[120] Rahman M.A., Yadab M.K., Ali M.M. (2024). Emerging role of extracellular pH in tumor microenvironment as a therapeutic target for cancer immunotherapy. Cells.

[121] Chiche J., Brahimi-Horn M.C., Pouyssegur J. (2010). Tumour hypoxia induces a metabolic shift causing acidosis: A common feature in cancer. J. Cell. Mol. Med..

[122] Wu J., Deng M., Ye F., Deng W., Wu Z., Zheng X., Song C., Zou Y., Tang H. (2026). Hijacking the bone niche: Mechanistic insights into bone metastasis in breast cancer. Bone Res..

[123] Kachler K., Andreev D., Thapa S., Royzman D., Giessl A., Karuppusamy S., Perez M.L., Liu M., Hofmann J., Gessner A. (2024). Acod1-mediated inhibition of aerobic glycolysis suppresses osteoclast differentiation and attenuates bone erosion in arthritis. Ann. Rheum. Dis..

[124] Sun Y., Li J., Xie X., Gu F., Sui Z., Zhang K., Yu T. (2021). Macrophage-osteoclast associations: Origin, polarization, and subgroups. Front. Immunol..

[125] Padua D., Zhang X.H.F., Wang Q., Nadal C., Gerald W.L., Gomis R.R., Massague J. (2008). TGFbeta primes breast tumors for lung metastasis seeding through angiopoietin-like 4. Cell.

[126] Trivedi T., Pagnotti G.M., Guise T.A., Mohammad K.S. (2021). The role of TGF-β in bone metastasis. Biomolecules.

[127] Li Z., Wang H. (2021). Molecular mechanisms of the SLC13A5 gene transcription. Metabolites.

[128] http://www.proteinatlas.org/ENSG00000158296-SLC13A3/cell+line.

[129] Lin H., Tison K., Du Y., Kirchhoff P., Kim C., Wang W., Yang H., Pitter M., Yu J., Liao P. (2024). Itaconate transporter SLC13A3 impairs tumor immunity via endowing ferroptosis resistance. Cancer Cell.

[130] Huard K., Brown J., Jones J.C., Cabral S., Futatsugi K., Gorgoglione M., Lanba A., Vera N.B., Zhu Y., Yan Q. (2015). Discovery and characterization of novel inhibitors of the sodium-coupled citrate transporter (NaCT or SLC13A5). Sci. Rep..

[131] Huard K., Gosset J.R., Montgomery J.I., Gilbert A., Hayward M.M., Magee T.V., Cabral S., Uccello D.P., Bahnck K., Brown J. (2016). Optimization of a dicarboxylic series for in vivo inhibition of citrate transport by the solute carrier 13 (SLC13) family. J. Med. Chem..

[132] Higuchi K., Kopel J.J., Sivaprakasam S., Jaramillo-Martinez V., Sutton R.B., Urbatsch I.L., Ganapathy V. (2020). Functional analysis of a species-specific inhibitor selective for human Na^+^-coupled citrate transporter (NaCT/SLC13A5/mINDY). Biochem. J..

[133] Quigley A., Kley J.T., Pautsch A., Kauschke S.G., Chu-Antypas A., Tessitore A., Strain-Damerell C., Shrestha L., Wiedenmayer D., Burgess-Brown N.A. (2026). Discovery and characterization of a nonacidic small-molecule inhibitor of the sodium-coupled dicarboxylate transporter NaCT. J. Med. Chem..

[134] Zahn G., Willmes D.M., El-Agroudy N.N., Yarnold C., Jarjes-Pike R., Schaerti S., Schreiter K., Gehrmann W., Wong A.K.C., Zordan T. (2022). A novel and cross-species active mammalian INDY (NaCT) inhibitor ameliorates hepatic steatosis in mice with diet-induced obesity. Metabolites.

[135] Zhang L., Hu W., Guo H., Sun Q., Xu X., Li Z., Qiu Z., Bian J. (2024). Discovery of highly potent solute carrier 13 member 5 (SLC13A5) inhibitors for the treatment of hyperlipidemia. J. Med. Chem..

[136] Mycielska M.E., Mohr M.T.J., Schmidt K., Drexler K., Rummele P., Haferkamp S., Schlitt H.J., Gaumann A., Adamski J., Geissler E.K. (2019). Potential use of gluconate in cancer therapy. Front. Oncol..

[137] Palmieri F., Stipani I., Quagliariello E. (1972). Kinetic study of the tricarboxylate carrier in rat liver mitochondria. Eur. J. Biochem..

[138] Aluvila S., Sun J., Harrison D.H., Walters D.E., Kaplan R.S. (2010). Inhibitors of the mitochondrial citrate transport protein: Validation of the role of substrate binding residues and discovery of the first purely competitive inhibitor. Mol. Pharmacol..

[139] Chen M., Li W., Tao Y., Hu C., Ge R., Kang S., Yue P., Man C.H., Wang L., Yan X. (2026). SLC25A1 reprograms mitochondrial and fatty acid metabolism to promote the progression of acute myeloid leukemia. Haematologica.

[140] Chen C., Bao Q., Huang H., Wang L., Jiang J., Liu H., Wang Y., Qing G., Fang Y. (2026). Mitochondrial citrate transport represents a metabolic liability in MYCN-amplified neuroblastoma. Mol. Ther..

[141] Wang H., Fei Y.J., Kekuda R., Yang-Feng T.L., Devoe L.D., Leibach F.H., Prasad P.D., Ganapathy V. (2000). Structure, function, and genomic organization of human Na^+^-dependent high-affinity dicarboxylate transporter. Am. J. Physiol. Cell Physiol..

[142] Runge B., Oktay H., Fucci I.J., Merten E.M., Tarasov S.G., Fan L., Monteiro D.C.F. (2026). Robust structural, kinetic and biophysical characterization of wild-type human ACOD1, selected mutants and their interaction with citraconate. J. Struct. Biol. X.

[143] Chen F., Elgaher W.A.M., Winterhoff M., Bussow K., Waqas F.H., Graner E., Pires-Afonso Y., Casares Perez L., de la Vega L., Sahini N. (2022). Citraconate inhibits ACOD1 (IRG1) catalysis, reduces interferon responses and oxidative stress, and modulates inflammation and cell metabolism. Nat. Metab..

[144] Toh J.Y.L., Zwe Y.H., Tan M.T.H., Gong Z., Li D. (2024). Sequential infection of human norovirus and Salmonella enterica resulted in higher mortality and ACOD1/IRG1 upregulation in zebrafish larvae. Microbes Infect..

